# Residue, dissipation and dietary intake risk assessment of tolfenpyrad in four leafy green vegetables under greenhouse conditions

**DOI:** 10.1016/j.fochx.2022.100241

**Published:** 2022-02-04

**Authors:** Tingting Lan, Guangqian Yang, Jianmin Li, Du Chi, Kankan Zhang

**Affiliations:** State Key Laboratory Breeding Base of Green Pesticide and Agricultural Bioengineering, Key Laboratory of Green Pesticide and Agricultural Bioengineering, Ministry of Education, Guizhou University, Guiyang 550025, China

**Keywords:** Tolfenpyrad, Green leafy vegetables, Greenhouse, Residue, Dissipation, Dietary intake risk

## Abstract

A novel and accurate analytical method for the determination of tolfenpyrad in four leafy green vegetables, *Brassica bara* L., *Spinacia oleracea* L., *Lactuca sativa* L. and *Brassica chinensis* L., was developed and applied to investigate the residue distribution and dietary risk under greenhouse conditions. The established approach was determined to be adequate, with recoveries of 79.2%–92.9% and relative standard deviations < 8%. Tolfenpyrad dissipated relatively rapidly in four leafy green vegetables. Terminal residues of tolfenpyrad were below 0.5 mg/kg (maximum residue limit for *Brassica bara* L. set by China) in leafy green vegetables collected 28 d after the last application. Due to risk quotient values < 100%, the residue levels of tolfenpyrad in leafy green vegetables collected 21 days after the last application were deemed safe for consumers. The results provide field data for the reasonable use and dietary risk assessment of tolfenpyrad in leafy green vegetables.

## Highlights

• A QuEChERS-GC–MS/MS method was used to detect tolfenpyrad in leafy green vegetables.
• Half-lives of tolfenpyrad were 2.0–6.8 d in greenhouse-grown leafy green vegetables.
• PHI of tolfenpyrad was suggested as 21 d in BCL and 28 d in BBL, SOL and LSL.
• The potential health risk of tolfenpyrad was acceptable in leafy green vegetables.

## Graphical abstract

**Figure:**
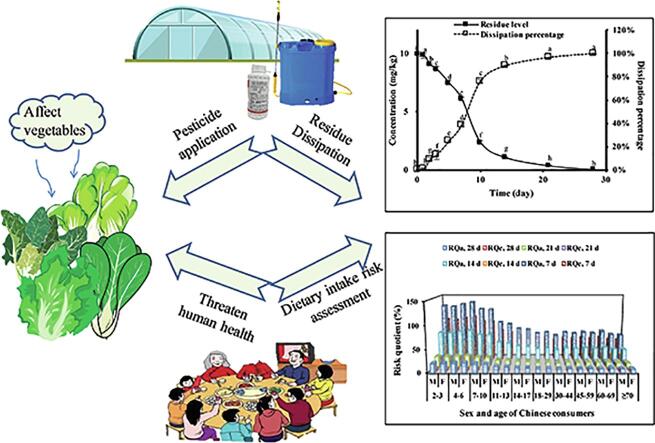

## Introduction

Leafy green vegetables, which include cruciferae (such as Chinese cabbage (*Brassica bara* L., BBL) and Shanghai green (*Brassica chinensis* L., BCL)); compositae (such as lettuce (*Lactuca sativa* L., LSL)); amaranthus (such as spinach (*Spinacia oleracea* L., SOL)) and other leafy green vegetables (Takahama et al., 2019), play an important role in people’s daily diet. Leafy green vegetables are rich in a variety of nutrients, including vitamins, proteins, and minerals, which are necessary for the human body (Li et al., 2021, Park et al., 2021). Because of the multitudinous bioactive components, leafy green vegetables play an important role in healing wounds and reducing the risk of chronic diseases (Farina et al., 2017, Li et al., 2021). For example, systemic research demonstrated that when the intake of leafy green vegetables increased, the potential risk of type 2 diabetes was reduced (Carter, Gray, Troughton, Khunti, & Davies, 2010). Because greenhouse is a relatively compact environment that limits the evaporation of water, weakens or blocks certain wavelengths of light, and produces a year-round plant growth agricultural ecosystem (Emekli, Kendirli, & Kurunc, 2010), the greenhouse cultivation approach is widely used to produce leafy green vegetables (Chen et al., 2021, Wang et al., 2020). In China, leafy green vegetables are planted in a wide range of locations under greenhouse conditions. However, a large number of greenhouse cultivated leafy green vegetables suffer from disease and pests every year, causing significant losses to the agriculture and food processing industry (Zhang et al., 2021). To control these damages, more than 800 kinds of pesticides are used in agricultural practices (Fan et al., 2013), among which the use of chemical pesticides is one of the most effective measures (Carneiro et al., 2013, Samsidar et al., 2018). Nonetheless, the indiscriminate use of pesticides leads to high concentrations of pesticide residue in agricultural products, posing a possible threat to consumer health (Liang et al., 2011). After spraying pesticides, greenhouse could limit the diffusion and photolysis of pesticides, and the residue levels of pesticides might be higher than those in open fields (Katsoulas, Boulard, Tsiropoulos, Bartzanas, & Kittas, 2012), which might cause more ecological and human health risks (Song et al., 2020). Combining pesticide abuse and potential risk factors, it is crucial to conduct research on the residue distribution, dissipation behavior and dietary intake risk assessment of pesticides on leafy green vegetables under greenhouse conditions.

Tolfenpyrad, whose mechanism of action is inhibiting complex I in the respiratory electron-transfer chain of mitochondria, is a new type of pyrazolamide insecticide. Tolfenpyrad could be effective against pests that are resistant to carbamate and organophosphate insecticides (Wimer et al., 2015, Yamaguchi et al., 2012). In China, tolfenpyrad is only registered for one leafy green vegetable (BBL) for the control of *Plutella xylostella* L., which is one of the most dominant pests in leafy green vegetable cultivation. According to the evaluation reports of tolfenpyrad (Committee, 2004), acute oral LD_50_ (lethal dose, 50%) values of tolfenpyrad for male and female rats were 386 and 150 mg/kg, respectively; acute dermal LD_50_ values were > 2000 and > 3000 mg/kg, respectively; and acute inhalation LC_50_ (lethal concentration, 50%) values were > 2.21 and > 1.5 mg/L, respectively. In a 90-day sub-chronic toxicity test, the maximum non-effect doses were < 0.91 mg/kg b.w./d in male rats and < 1.01 mg/kg b.w./d in female rats. Moreover, tolfenpyrad could induce chromosomal aberrations in cultured Chinese hamster cells, and tolfenpyrad was classified as a moderately toxic insecticide. However, the prolonged application of pesticides has inevitably caused concerns about the residues in leafy green vegetables and the environment, which could lead to potential threat toward human health (Dong et al., 2018). To monitor the residue concentration in leafy green vegetables and evaluate the threat to humans, a suitable detection approach and proper field trials have been proposed as the main process. According to previous studies, a few detection approaches have been applied to determine the concentration of tolfenpyrad in crops, such as liquid chromatography with tandem mass spectrometry (LC–MS/MS) and gas chromatography with tandem mass spectrometry (GC–MS/MS) (Kmellár et al., 2008, Lim et al., 2018, Soliman et al., 2019, Wang et al., 2012, Wang et al., 2014). In addition, the residues of tolfenoyrad have been evaluated on tea and circus under open field conditions. Dong et al. (2018) reported that the dissipation half-life value of tolfenpyrad was 14.1 d in circus and the dietary intake risk was acceptable for human consumption. Bai et al. (2021) found that tolfenpyrad dissipated fast in tea (half-lives of 4.3–7.3 d) and the potential health risk induced by this insecticide in tea were not significant for consumers. However, information related to the residual analysis and dietary risk of tolfenpyrad in leafy green vegetables, especially under greenhouse conditions, is lacking.

In this work, the dissipation, residue concentrations and dietary intake risk of tolfenpyrad were investigated in four leafy green vegetables under greenhouse conditions in Guiyang, China, and based on the existing reports, this paper aimed at: (1) to develop and validate an analytical approach to determine the concentrations of tolfenpyrad in four leafy green vegetables (BBL, SOL, LSL and BCL); (2) to investigate the dissipation of tolfenpyrad in four leafy green vegetables greenhouse cultivation systems; (3) to assess the terminal residue levels of tolfenpyrad in four leafy green vegetables; and (4) to provide some useful data for guiding the proper use of tolfenpyrad in four leafy green vegetables in China using the dietary intake risk assessment method.

## Materials and methods

### Chemical reagents and materials

Tolfenpyrad (98.0% purity) was purchased from Beijing J&K Scientific Co., Ltd. (Beijing, China). The commercial formulation included suspension concentrate (SC) containing 15% tolfenpyrad and was provided by Hailir Pesticides and Chemicals Group (Qingdao, China). Acetonitrile (ACN), acetone, methanol (MeOH), ethyl acetate (EA), acetic acid (AA), sodium chloride (NaCl) and anhydrous magnesium sulfate (MgSO_4_) were purchased from Chengdu Jinshan Chemical Reagent Company (Chengdu, China). Primary secondary amine (PSA), octadecylsilane (C_18_) and graphitized carbon black (GCB) were purchased from Bonna-Agela Technologies Co., Ltd. (Tianjin, China). Syringe filters (0.22 µm, nylon) were obtained from PeakSharp Technologies (Beijing) Co., Ltd. (Beijing, China).

The stock solution was prepared by weighing 0.0101 g of tolfenpyrad standard into a 100 mL volumetric flask and was then dissolved using chromatographic grade acetone at a concentration of 101 µg/mL. The standard working solutions at concentrations of 0.005, 0.05, 0.1, 0.5, 1, 5, and 10 µg/mL were diluted with acetone. Matrix-matched standard solutions of tolfenpyrad were prepared in blank BBL, SOL, LSL and BCL samples for calibration and quantitation. All solutions were stored at −20 °C in the dark and were stable for one month.

### Instrumentation

Analysis was performed using a TRACE 1310 gas chromatography (GC) coupled with a TSQ 8000 Evo triple quadrupole mass spectrometer (Thermo Fisher Scientific, Waltham, USA). Chromatographic separation was conducted using an Agilent J&W DB-35 ms column (30 m × 0.25 mm, 0.25 µm, Agilent Technologies, CA, USA). GC parameters were set as follows; carrier gas: helium (99.999%), flow rate: 1.2 mL/min, injection volume: 1 μL, inlet temperature: 240 °C, ion source temperature: 300 °C, transmission line temperature: 280 °C, programmed temperature vaporization (PTV) injection port mode: programmed temperature rise without splitting, temperature program: initial temperature was set at 100 °C, held for 0.5 min, then raised to 220 °C at 20 °C/min, held for 1 min, increased to 300 °C at 40 °C/min, held for 3 min. The MS/MS optimized parameters are shown in Table S1 (Supplementary information).

### Greenhouse field trials

A field experiment was designed according to the “Guidelines on pesticide residue trials NY/T 788–2018 (China)”. The experiments were conducted from June 2020 to September 2020 in Guizhou, China on four leafy green vegetables under greenhouse conditions. The greenhouse was divided into a blank control plot and five test plots. Blank control plots were used without tolfenpyrad application, and every plot had a set size of 50 m^2^ (5 m × 10 m) and a 0.5 m wide buffer zone. To investigate the dissipation dynamics and terminal residue of tolfenpyrad on BBL, SOL, LSL and BCL, tolfenpyrad (15%, SC) formulations were applied in two doses (67.5 and 112.5 g a.i./ha) with two spraying times of either of once or twice (interval: 7 d). To understand the dissipation pattern of tolfenpyrad, fresh leafy green vegetable samples were randomly collected at 0 (2 h), 1, 2, 3, 5, 7, 10, 14, 21, and 28 d after the last spraying in every treatment. BBL, SOL, LSL and BCL samples were gathered randomly at 7, 14, 21 and 28 d after the last application to monitor the terminal residues of tolfenpyrad. Each treatment was conducted in triplicate, and fresh samples (approx. 2 kg of each vegetable type) were randomly collected in each plot. All samples were sent to the laboratory within 5 h after collection and were then homogenized and stored at −20 °C before analysis.

### Sample pretreatment

Aliquots of 10 ± 0.02 g of leafy green vegetable samples were weighed and transferred to a 50 mL centrifuge tube. Twenty milliliters of ACN were added as the extractant, and 4 g of anhydrous MgSO_4_ and 2 g of NaCl were added as the dehydrant and salting-out agents, respectively. The mixture was vortexed at a relative centrifugal force (RCF) of 1677×*g* for 3 min and centrifuged at 4025×*g* for 5 min. One milliliter of the supernatant was collected and evaporated at 40 °C. The residues were dissolved in 1 mL of acetone and then transferred into a 2 mL centrifuge tube containing 100 mg of PSA. After vortexing at 1677×*g* for 0.5 min and centrifuging at 6708×*g* for 2 min, the supernatant was filtered through a 0.22 μm nylon syringe filter before GC–MS/MS analysis.

### Method validation

Validation parameters, including linearity, matrix effect (ME), recovery rate, limit of detection (LOD), limit of quantification (LOQ), accuracy and precision, were verified to determine the amount of tolfenpyrad in leafy green vegetables (Commission, 2019). Calibration curves plotted by peak areas vs. pesticide concentrations in solvent and matrices were used to evaluate the linearity (Hepsağ & Kizildeniz, 2021). ME is caused by other components present in the matrix, which could inhibit or enhance the analytical signal of the target compound, resulting in a lower or higher recovery rate (Matadha, Mohapatra, & Siddamallaiah, 2021). The slope ratio of the matrix-matched and solvent calibration curve was determined to evaluate the ME of tolfenpyrad in each leafy green vegetable (Zou et al., 2016). LODs and LOQs of tolfenpyrad were determined at a signal-to-noise ratio (S/N) of 3:1 and the lowest spiked level of each matrix (Sharma et al., 2018). The accuracy and precision of the developed method were assessed by the results (recovery and relative standard deviation (RSD)) for the intra-day and inter-day recovery assays at four spiked concentrations (0.01, 0.1, 1, and 10 mg/kg) with five replicates on three different days for tolfenpyrad in every leafy green vegetable (Qin et al., 2020, Tsochatzis et al., 2010).

### Acute and chronic dietary intake risk assessment for Chinese consumers

Dietary intake risk was assessed to determine the health risk and quantify the repercussions after pesticide application. The acute risk quotient (RQ_a_), used to assess the acute dietary intake risk was calculated by the equations of NESTI = FI × HR/b.w. and RQ_a_ = NESTI/ARfD × 100%. The chronic risk quotient (RQ_c_), applied to the evaluation of chronic dietary intake risk, was calculated by the equations of IEDI = FI × STMR/b.w. and RQ_c_ = IEDI/ADI × 100% (FAO and WHO, 2011). In these equations, NESTI (mg/kg b.w.) is the national estimated short-term intake, FI (g/day) is the food intake referring to the GEMS (Global Environment Monitoring System)/food cluster diets, HR (mg/kg) is the highest residue level, b.w. (kg) is the body weight, ARfD (mg/kg b.w.) is the acute reference dose, IEDI (mg/kg b.w.) is the international estimated daily intake, STMR (mg/kg) is the median residue level, and ADI (mg/kg b.w.) is the acceptable daily intake. RQ > 100% indicates that the risk for humans is unacceptable, and RQ < 100% indicates a minimal risk to humans and is deemed acceptable (Song et al., 2020).

### Data and statistical analyses

Two first-order kinetic equations, *C_t_* = *C*_0_ *e*^-^*^kt^* and *t*_1/2_ = ln2/*k*, were used to determine the dissipation dynamics of tolfenpyrad in four leafy green vegetables under greenhouse conditions. In these equations, *C*_t_ (mg/kg) is the concentration at time *t* (d) after application, *C*_0_ (mg/kg) is the initial concentration, *k* (d^-1^) is the dissipation rate constant, and *t*_1/2_ (d) is the half-life (Qin et al., 2020). Excel 2010 (Microsoft Corporation, Washington, D. C., USA) was used to collate the data. Duncan’s multiple range test (*P* < 0.01) was conducted in SPSS 24.0 (SPSS Inc., Chicago, USA) for statistical analysis. The dissipation parameters and terminal residual concentrations are shown as the average values of three replicate data points after statistical analysis.

## Results and discussion

### Optimization of the determination method

To develop a better detection approach for tolfenpyrad, three different types of chromatographic columns, including Agilent J&W DB-35 ms GC column (30 m × 0.25 mm, 0.25 µm); Agilent J&W DB-5 ms GC column (30 m × 0.25 mm, 0.25 µm, Agilent Technologies, CA, USA); and Thermo Scientific™ TraceGOLD™ TG-5MS GC column (30 m × 0.25 mm, 0.25 µm, Thermo Fisher Scientific, MA, USA), were evaluated for the detection of telfonpyrad by GC–MS/MS. As shown in Fig. S1 (Supplementary information), the optimized column was an Agilent J&W DB-35 ms GC column, which exhibited a relatively higher resolution, a shorter retention time and a better peak shape for tolfenpyrad. In addition to the chromatographic column, the temperature program is another crucial factor influencing the separation of tolfenpyrad. The chromatograms obtained from two programs (Fig. S2, Supplementary information) illustrated that the peak area and retention time were more adequate for detecting tolfenpyrad when the temperature program listed in Section 2.2 was applied.

To obtain a better recovery rate of tolfenpyrad and to more effectively remove impurities from the leafy green vegetable samples, different extraction methods, extraction times, extraction solutions, volumes of extractant and purification sorbents were evaluated. In Fig. S3 (Supplementary information), two extraction methods (vortex and ultrasound) and three extraction times (3, 5 and 10 min) were selected. Due to the relatively lower standard deviations (SDs) and higher recoveries, the extraction efficiency and stability of the vortex were found to be significantly better than those of the ultrasound (*P* < 0.01). To save time, vortexing for 3 min was chosen as the optimal extraction method and extraction time for tolfenpyrad in four leafy green vegetables. Five extraction solutions, including MeOH, EA, ACN, ACN (0.1% AA), and ACN (1% AA), were applied. The data in Fig. S4 (Supplementary information) showed that the recovery of tolfenpyrad extracted by ACN was significantly higher (*P* < 0.01), whereas the other four extractants provided unsatisfactory extraction efficiencies. To simplify the procedure and protect the environment, ACN was selected as the optimized extractant. Furthermore, different volumes (10, 20 and 25 mL) of ACN were assessed, and the results (Fig. S5, Supplementary information) demonstrated that among them, the optimal volume of ACN was 20 mL because it had significantly better recovery (near 100%). Finally, different combinations of sorbents (50 mg C_18_, 100 mg C_18_, 50 mg PSA, 100 mg PSA, 50 mg GCB, 100 mg GCB and 50 mg PSA + 50 mg C_18_) were screened to purify leafy green vegetables more efficiently. In Fig. S6 (Supplementary information), when 100 mg PSA was applied, the recoveries of tolfenpyrad in the four leafy green vegetables (99.8% for BBL and SOL, 94.5% for LSL and 91.8% for BCL) were significantly better than those of the other sorbents (*P* < 0.01). Therefore, 100 mg PSA was selected as the optimized purification sorbent.

### Method validation

**Fig. 1:**
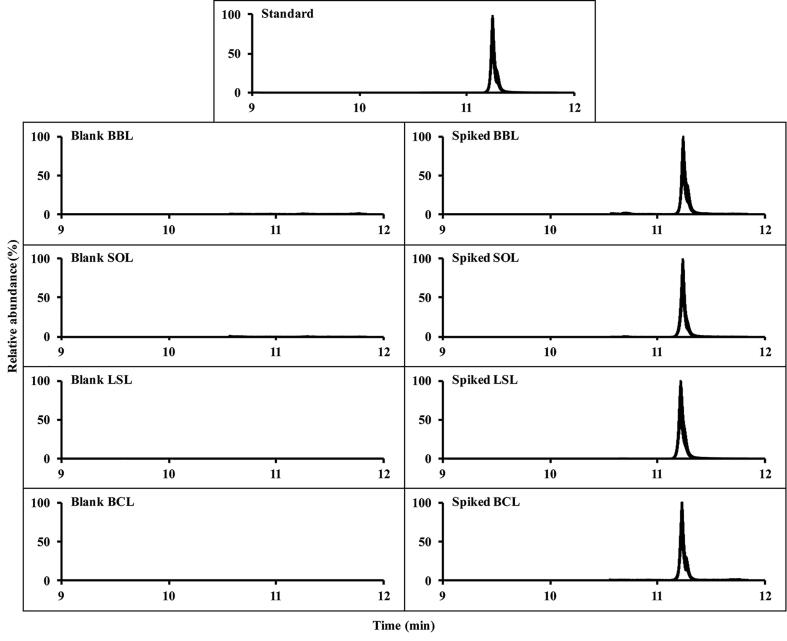
GC–MS/MS chromatograms of tolfenpyrad in standard solution (0.05 µg/mL), blank BBL, SOL, LSL, and BCL samples, as well as spiked BBL, SOL, LSL and BCL samples (0.01 mg/kg). (BBL: *Brassica bara* L.; SOL: *Spinacia oleracea* L.; LSL: *Lactuca sativa* L.; and BCL: *Brassica chinensis* L.).

Typical GC–MS/MS chromatograms of tolfenpyrad in standard solution (0.05 µg/mL), in blanks, and in spiked (0.01 mg/kg) leafy green vegetable samples are shown in Fig. 1. No interference occurred, therefore adequate qualitative and quantitative analyses were obtained according to the above established approach. Based on the SANTE/12682/2019 document (Commission, 2019), satisfactory determination coefficients (*R*^2^ > 0.999) were achieved (Table S2, Supplementary information), which induced good linearities of the solvent and matrix-matched calibration curves for tolfenpyrad. Since the respective ME values of BBL, SOL, LSL and BCL were 0.87, 0.72, 0.78 and 0.91, a matrix suppression effect was observed, and matrix-matched calibration curves were used for the residual calculation of tolfenpyrad. The LODs and LOQs of the developed method for tolfenpyrad were 0.003 and 0.01 mg/kg, respectively, in the four leafy green vegetables. The recovery rates and RSDs were calculated to assess the method’s accuracy and precision. In Table 1, the average recovery rates of tolfenpyrad ranged from 79.2% to 92.9% in leafy green vegetable samples with intra-day RSDs of 1.1% to 7.1% and inter-day RSDs of 2.7% to 7.5%. The results indicate that the developed residual analytical approach is suitable for the extraction and detection of tolfenpyrad in four leafy green vegetables.

**Table 1:** Recoveries and relative standard deviations (RSDs) of tolfenpyrad in different leafy green vegetables.

Matrix	Spiked level (mg/kg)	Intra-day average recovery, RSD (%, *n* = 5)			Inter-day average recovery, RSD (%, *n* = 15)
		Day 1	Day 2	Day 3	
BBL	0.01	85.5, 6.6	84.4, 1.3	88.1, 2.3	86.0, 4.2
	0.1	82.4, 2.3	87.0, 4.0	81.6, 2.6	83.7, 4.1
	1	89.2, 6.9	81.0, 3.0	83.2, 2.2	84.5, 6.1
	10	82.6, 2.2	84.2, 1.8	82.9, 4.6	83.2, 3.0
SOL	0.01	88.4, 3.1	81.8, 2.5	83.5, 5.2	84.6, 4.9
	0.1	81.4, 3.6	82.2, 3.5	79.2, 4.7	80.9, 4.0
	1	85.3, 2.7	83.2, 1.8	81.9, 2.8	83.5, 2.9
	10	83.5, 6.8	83.7, 7.1	92.9, 2.8	86.7, 7.5
LSL	0.01	90.8, 5.8	88.5, 3.3	88.4, 2.0	89.2, 4.0
	0.1	89.4, 3.4	91.2, 6.7	82.7, 5.0	87.8, 6.5
	1	87.7, 1.8	85.4, 4.4	81.6, 5.1	84.9, 4.8
	10	81.7, 1.6	84.2, 3.1	81.6, 2.2	82.5, 2.7
BCL	0.01	81.3, 3.1	87.2, 2.6	90.7, 3.8	86.4, 5.5
	0.1	84.2, 3.6	84.8, 3.8	80.7, 2.1	83.2, 3.8
	1	85.3, 2.2	85.0, 4.3	87.1, 3.8	85.8, 3.5
	10	81.2, 1.1	85.4, 5.1	83.4, 2.5	83.3, 3.8

BBL: *Brassica bara* L.; SOL: *Spinacia oleracea* L.; LSL: *Lactuca sativa* L.; and BCL: *Brassica chinensis* L.

### Dissipation dynamics and terminal residue of tolfenpyrad in four leafy green vegetables under greenhouse conditions

Under greenhouse conditions, the dissipation behaviors of tolfenbyrad in BBL, SOL, LSL, and BCL were investigated. The residue concentrations and dissipation percentages of tolfenpyrad in four leafy green vegetables under greenhouse conditions are listed in Figs. S7-S10 (Supplementary information). The initial concentrations in most samples were positively related to the spraying dose and spraying time. For example, when the spraying dose was 67.5 g a.i./ha, the initial residue level (7.95 mg/kg) of tolfenpyrad at spraying twice was as high (9.09 mg/kg) comparted to spraying once in LSL (Fig. S9, Supplementary information). When the leafy green vegetables were sprayed twice, the initial concentration (11.85 mg/kg) at a dose of 67.5 g a.i./ha was lower than when samples were sprayed once (17.06 mg/kg) at a dose of 112.5 g a.i./ha in BCL (Fig. S10, Supplementary information). As seen from the dissipation percentages in the Supplementary information, tolfenpyrad dissipated significantly with time (*P* < 0.01). At 28 d after the last application, the residues of tolfenpyrad decreased by more than 90% in all of the treatments. The dissipation patterns of tolfenpyrad in the four green leafy vegetables followed a first-order kinetic model with correlation coefficients of 0.8454–0.9823 (Table 2). In different treatments, the half-lives of tolfenpyrad were 4.0–6.8 d, 5.3–6.6 d, 3.5–4.5 d, and 2.0–3.1 d in BBL, SOL, LSL and BCL, respectively. The half-lives of the four green leafy vegetables under greenhouse conditions were shorter than those for fresh tea leaves (4.3–7.3 d, Bai et al., 2021) and citrus plants (14.1 d, Dong et al., 2018) under open field conditions. Similar results were also reported in which imidaclothiz and pyridaben dissipated faster in greenhouse-grown *Barrassica campestris* spp*.* samples than in open field samples (Tang et al., 2021). The dissipation of pesticides in crops normally occurs through multiple pathways, including volatilization, photolysis, hydrolysis, microbial degradation, metabolic conversion, transportation to other sites and release into the air through respiration (Wang et al., 2018; Di et al., 2021). Among them, the main dissipation routes of pesticides are mediated by metabolic enzyme in crops and microbial catabolism induced by the microorganisms in soil environment (Chen et al., 2020, Li et al., 2013). Some previous papers demonstrated that cytochrome P450 3A4 mediated the dissipation of metconazole in tomato leaves and *Sphingobacterium multivorum* B-3 (a soil-originated organism) degraded hexaconazole (An et al., 2020, Zhuang et al., 2018). Thus, the dissipation of tolfenpyrad in leafy green vegetables was possibly related to mediation through metabolic enzyme and/or microbial catabolism. The statistical analysis results (Table 2) demonstrated that the dissipation rate of tolfenpyrad was significantly influenced by the spraying dose and spraying time (*P* < 0.01). For BBL, SOL and BCL, the half-lives of tolfenpyrad increased with increasing spraying dose and spraying time. However, the half-lives of tolfenpyrad for LSL decreased when the spraying dose increased. Several factors might contribute to the dissipation fate of pesticides in vegetables. However, under similar environmental conditions and application method, the vegetable characteristics, including morphology, growth rate and growth stage at treatment, were most important (Ramezani & Shahriari, 2015). For instance, the residue levels of pesticides in vegetables could be affected by the shape of vegetables when pesticides were applied to foliage (Ripley, Ritcey, Harris, Denommé, & Lissemore, 2003). Because four leafy vegetables were cultivated under greenhouse conditions and treated at the same approach, these differences among the dissipation rates of tolfenpyrad in leafy green vegetables might be related to the variety, shape and growth rate.

**Table 2:** Dissipation parameters of tolfenpyrad in different leafy green vegetables at different doses and spraying times.

Matrix	Dose (g a.i./ha)	Spraying time	*R*^2^	*P*_1_	*K (*d^-1^*)*	*P*_2_	*t*_1/2_ (d)	*P*_3_
BBL	67.5	1	0.9695 ± 0.0034^a^	0.007	0.1680 ± 0.0016^a^	＜0.001	4.1 ± 0.1^c^	＜0.001
		2	0.9319 ± 0.0069^b^		0.1214 ± 0.0036^b^		5.7 ± 0.2^b^	
	112.5	1	0.9732 ± 0.0066^a^		0.1713 ± 0.0047^a^		4.0 ± 0.1^c^	
		2	0.9713 ± 0.0208^a^		0.1012 ± 0.0039^c^		6.8 ± 0.3^a^	
SOL	67.5	1	0.9305 ± 0.0126^b^	＜0.001	0.1304 ± 0.0009^a^	＜0.001	5.3 ± 0.1^b^	＜0.001
		2	0.9774 ± 0.0090^a^		0.1049 ± 0.0058^b^		6.6 ± 0.4^a^	
	112.5	1	0.9724 ± 0.0045^a^		0.1100 ± 0.0033^b^		6.3 ± 0.2^a^	
		2	0.9823 ± 0.0053^a^		0.1089 ± 0.0027^b^		6.4 ± 0.2^a^	
LSL	67.5	1	0.9355 ± 0.0104^a^	＜0.001	0.1627 ± 0.0038^b^	＜0.001	4.3 ± 0.1^a^	＜0.001
		2	0.9645 ± 0.0041^a^		0.1526 ± 0.0017^b^		4.5 ± 0.1^a^	
	112.5	1	0.9689 ± 0.0032^a^		0.1962 ± 0.0053^a^		3.5 ± 0.1^b^	
		2	0.8454 ± 0.0232^b^		0.2009 ± 0.0162^a^		3.5 ± 0.3^b^	
BCL	67.5	1	0.9817 ± 0.0011^a^	0.002	0.3541 ± 0.0092^a^	＜0.001	2.0 ± 0.1^c^	＜0.001
		2	0.9602 ± 0.0074^ab^		0.2989 ± 0.0077^b^		2.3 ± 0.1^b^	
	112.5	1	0.9323 ± 0.0115^bc^		0.3300 ± 0.0077^a^		2.1 ± 0.1^bc^	
		2	0.9171 ± 0.0233^c^		0.2263 ± 0.0147^c^		3.1 ± 0.2^a^	

Data are expressed as average values ± SD. *P*_1_, *P*_2_ and *P*_3_ represent *R*^2^, *K* and *t*_1/2_, respectively, different lower case letters indicate statistical significance between different treatments for each leafy green vegetable by Duncan’s multiple range test (*P* < 0.01). BBL: *Brassica bara* L.; SOL: *Spinacia oleracea* L.; LSL: *Lactuca sativa* L.; and BCL: *Brassica chinensis* L.

The terminal residues of tolfenpyrad in four green leafy vegetables are shown in Table 3. With spraying doses of 67.5–112.5 g a.i./ha and spraying times of once or twice, the residues of tolfenpyrad significantly decreased with increasing sampling interval (*P* < 0.01). For example, the concentrations of tolfenpyrad in BBL were 1.42–3.50 mg/kg at intervals of 7 d, 0.44–1.99 mg/kg at 14 d, 0.11–0.65 mg/kg at 21 d, and 0.03–0.50 mg/kg at 28 d. Concentrations in SOL were 2.50–5.11 mg/kg at intervals of 7 d, 0.76–1.76 at 14 d, 0.61–0.99 mg/kg at 21 d and 0.08–0.40 mg/kg at 28 d, respectively. Terminal residue data are always used to recommend the safety preharvest interval (PHI) and the maximum residue limit (MRL) for pesticides on crops (Dong et al., 2021). The MRL value of tolfenpyrad in BBL was 0.5 mg/kg, as set by the National Health and Family Planning Commission and Ministry of Agriculture of the People's Republic of China (2020). For BCL samples collected at an interval of 21 d, the concentrations of tolfenpyrad were 0.01–0.17 mg/kg and were lower than the MRL set by China, which indicates that 21 d could be recommended as the PHI of tolfenpyrad for BCL under greenhouse conditions. However, the tolfenpyrad concentrations in the other three green leafy vegetables were higher than 0.5 mg/kg at an interval of 21 d (0.61–0.99 mg/kg) and lower than the MRL at an interval of 28 d (<0.01–0.50 mg/kg). Therefore, the recommended PHI of tolfenpyrad was 28 d for BBL, SOL and LSL under greenhouse conditions.

**Table 3:** Terminal residue concentrations of tolfenpyrad in different leafy green vegetables.

Dose (g a.i./ha)	Spraying time	Interval (day)	Terminal residue (mg/kg)							
			BBL	*P*_1_	SOL	*P*_2_	LSL	*P*_3_	BCL	*P*_4_
67.5	1	7	1.42 ± 0.04^c^	<0.001	2.50 ± 0.29^d^	<0.001	2.16 ± 0.09^d^	<0.001	1.96 ± 0.09^c^	<0.001
		14	0.57 ± 0.08^d^		0.76 ± 0.03^hi^		0.72 ± 0.01 ^fg^		0.08 ± 0.00^d^	
		21	0.20 ± 0.03^de^		0.75 ± 0.04^ij^		0.16 ± 0.01 ^h^		0.01 ± 0.00^d^	
		28	0.03 ± 0.00^f^		0.08 ± 0.01 ^l^		0.16 ± 0.02 ^h^		<0.01^d^	
	2	7	1.50 ± 0.14^c^		2.89 ± 0.19^c^		6.49 ± 0.75^b^		1.70 ± 0.06^c^	
		14	0.72 ± 0.01^d^		1.76 ± 0.20^e^		1.69 ± 0.02^e^		0.32 ± 0.00^d^	
		21	0.50 ± 0.03^ef^		0.61 ± 0.07^ijk^		0.34 ± 0.03^gh^		0.11 ± 0.01^d^	
		28	0.06 ± 0.01^f^		0.26 ± 0.06^kl^		0.18 ± 0.01 ^h^		<0.01^d^	
112.5	1	7	1.97 ± 0.22^b^		4.32 ± 0.22^b^		6.08 ± 0.18^b^		7.76 ± 0.18^a^	
		14	0.44 ± 0.04^de^		1.27 ± 0.07 ^fg^		1.07 ± 0.08^f^		0.73 ± 0.03^d^	
		21	0.11 ± 0.02^f^		0.88 ± 0.06^hi^		0.36 ± 0.03^gh^		0.01 ± 0.00^d^	
		28	0.07 ± 0.01^f^		0.31 ± 0.04^kl^		0.04 ± 0.01 ^h^		<0.01^d^	
	2	7	3.50 ± 0.37^a^		5.11 ± 0.31^a^		8.05 ± 0.21^a^		7.70 ± 0.49^a^	
		14	1.99 ± 0.20^b^		1.60 ± 0.08^ef^		3.69 ± 0.05^c^		5.01 ± 1.33^b^	
		21	0.65 ± 0.13^d^		0.99 ± 0.15^gh^		0.87 ± 0.10^f^		0.17 ± 0.01^d^	
		28	0.50 ± 0.06^d^		0.40 ± 0.03^jkl^		0.02 ± 0.01 ^h^		0.03 ± 0.02^d^	

Data are expressed as average values ± SD. *P*_1_, *P*_2_, *P*_3_ and *P*_4_ represent BBL, SOL, LSL and BCL, respectively, different lower case letters indicate statistical significance between different treatments for each leafy green vegetable by Duncan’s multiple range test (*P* < 0.01). BBL: *Brassica bara* L.; SOL: *Spinacia oleracea* L.; LSL: *Lactuca sativa* L.; and BCL: *Brassica chinensis* L.

### Dietary intake risk assessment of tolfenpyrad in four leafy green vegetables for Chinese consumers

Acute dietary intake risk assessment and chronic dietary intake risk assessment are two crucial points to ensure dietary safety after the application of pesticides in crops (Tang et al., 2021). Based on a recent study (Qin et al., 2020), a new evaluation model involving the dietary intake of humans of different body weights, sexes and ages was conducted for the dietary intake risk assessment of tolfenpyrad for Chinese consumers in four leafy green vegetables. According to the dietary intake report of Chinese residents (Jin, 2008), the body weights of Chinese consumers were 12.3–64.9 kg and the daily intake of dark vegetables ranged from 39.6 to 99.5 g/d. The ADI and ARfD of tolfenpyrad, set by the Joint FAO/WHO Meeting on Pesticide Residues report (JMPR, 2016), were 0.006 and 0.01 mg/kg b.w., respectively. At all four sampling intervals, the STMRs and HRs of tolfenpyrad were 0.06–1.70 and 0.64–3.93 mg/kg in BBL, 0.29–3.61 and 0.43–5.40 mg/kg in SOL, 0.09–6.12 and 0.19–8.26 mg/kg in LSL, and 0.01–4.69 and 0.08–8.25 mg/kg in BCL, respectively.

**Fig. 2:**
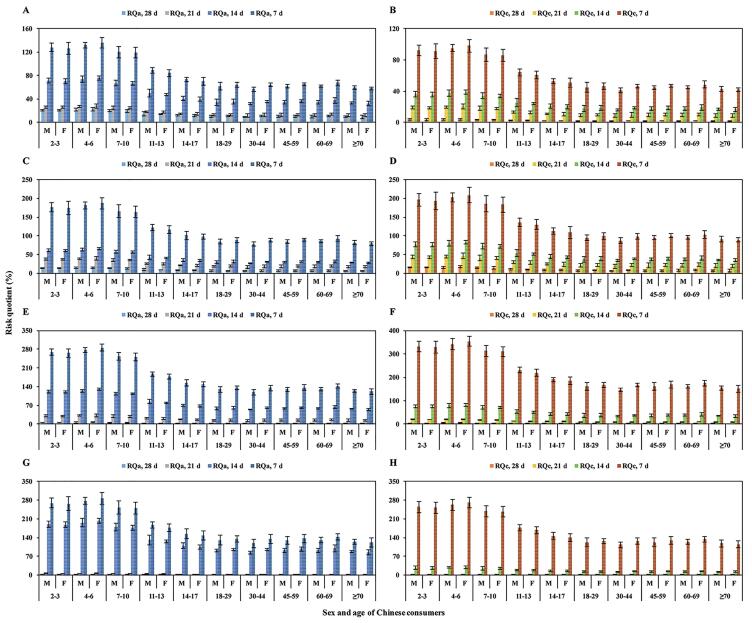
Acute risk (RQ_a_) and chronic risk (RQ_c_) of tolfenpyrad in BBL (A and B), SOL (C and D), LSL (E and F) and BCL (G and H) collected 28 d, 21 d, 14 d and 7 d after the last application in all four treatments for different Chinese consumer groups. (F: female; M: male; BBL: *Brassica bara* L.; SOL: *Spinacia oleracea* L.; LSL: *Lactuca sativa* L.; and BCL: *Brassica chinensis* L.).

To assess the dietary intake risk of tolfenpyrad in BBL, the calculated NESTIs and RQ_a_s for different groups of Chinese consumers were 0.0010–0.0022 mg/kg b.w. and 9.3%–22.4% for intervals of 28 d, 0.0012–0.0028 mg/kg b.w. and 11.6%–27.9% for 21 d, 0.0032–0.0076 mg/kg b.w. and 31.7%–76.1% for 14 d, and 0.0057–0.0136 mg/kg b.w. and 56.7%–136% for 7 d, respectively. The corresponding IEDIs and RQ_c_s were 0.0001–0.0002 mg/kg b.w. and 1.6%-3.8% for 28 d, 0.0005–0.0012 mg/kg b.w. and 8.5%-20.4% for 21 d, 0.0010–0.0023 mg/kg b.w. and 16.1%-38.7% for 14 d, and 0.0025–0.0059 mg/kg b.w. and 40.9%-98.2% for 7 d, respectively (Fig. 2A and B). Due to the RQ assessment guidelines (FAO and WHO, 2011), the acute and chronic dietary intake risk posed by tolfenpyrad in BBL samples collected at 28 d, 21 d, and 14 d after the last application were acceptable for the health of different groups of Chinese consumers. Similar results (Fig. 2C and D) were observed in the risk assessment trials of tolfenpyrad in SOL, where the acute and chronic RQ values (6.3%–82.1%) were < 100%. However, the RQ_a_s (0.1%–6.6%) and RQ_c_s (1.4%–32.9%) of tolfenpyrad were lower than 100% only in LSL (Fig. 2E and F) and BCL (Fig. 2G and H) at intervals of 28 d and 21 d. The results indicated that the dietary intake risk of tolfenpyrad for Chinese consumers could be accepted in LSL and BCL samples collected 28 d and 21 d after the last application in all four treatments under greenhouse conditions.

Differences between several age and sex groups of Chinese consumers were also investigated in the dietary intake risk of tolfenpyrad in four leafy green vegetables. Possibly because of their lighter body weight, the acute and chronic RQs for children under 10 years old were higher than those for other consumers. For example, in Table S3 (Supporting information), the RQ_a_s for 2- to 10-year-old Chinese consumers were 19.6%–22.4%, whereas for other groups, the RQ_a_s ranged from 9.3% to 14.6% for BBL. In summary, the acute and chronic dietary intake risks posed by tolfenpyrad were acceptable for different groups of Chinese consumers after the application of this insecticide and provided further evidence that the PHI was 21 d for BCL and 28 d for the other three leafy green vegetables.

## Conclusion

In this paper, an efficient analytical approach based on a simple pretreatment method was developed for the determination of tolfenpyrad in four leafy green vegetables by GC–MS/MS method. According to the guidelines of the European Commission (2019), the approach was suitable for the residue analysis of tolfenpyrad with linearity (*R*^2^ > 0.999), accuracy (recovery of 79.2%–92.9%) and precision (RSDs < 8%). Field trials with four leafy green vegetables cultivated under greenhouse conditions were carried out following the guidelines on pesticide residue trials. Possibly due to the metabolic enzyme and/or microbial catabolism, the dissipation of tolfenpyrad were relatively fast in four greenhouse-grown leafy green vegetables with the half-lives of 2.0–6.8 d, which might lead to the low potential risk in the greenhouse ecosystem. The terminal residues of tolfenpyrad in BBL, SOL and LSL at a PHI of 28 d were 0.03–0.50 mg/kg and those in BCL at a PHI of 21 d were 0.01–0.17 mg/kg, which were less than the MRL in BBL set by China (0.50 mg/kg). The acute and chronic dietary risk assessment with different groups of Chinese consumers (RQ_a_ and RQ_c_ < 100%) revealed no potential risk to human health posed by tolfenpyrad in the four leafy green vegetables collected 21 d after the last application under greenhouse conditions. This work provided some valuable information to guide the proper and safe application of tolfenpyrad in leafy green vegetables under greenhouse conditions and conducted a preliminary risk assessment of tolfenpyrad for leafy green vegetable consumption in China.

## CRediT authorship contribution statement

**Tingting Lan:** Formal analysis, Methodology, Visualization, Writing – original draft. **Guangqian Yang:** Investigation, Validation. **Jianmin Li:** Formal analysis. **Du Chi:** Software. **Kankan Zhang:** Conceptualization, Resources, Funding acquisition, Project administration, Supervision, Writing – review & editing.

## Declaration of Competing Interest

The authors declare that they have no known competing financial interests or personal relationships that could have appeared to influence the work reported in this paper.
