# Supplementary figures and images for: Residue, dissipation and dietary intake risk assessment of tolfenpyrad in four leafy green vegetables under greenhouse conditions
Source: Food Chem X. 2022 Feb 4;13:100241. doi: 10.1016/j.fochx.2022.100241 (PMC9040032; doi:10.1016/j.fochx.2022.100241)

RT: 0.00 - 11.84

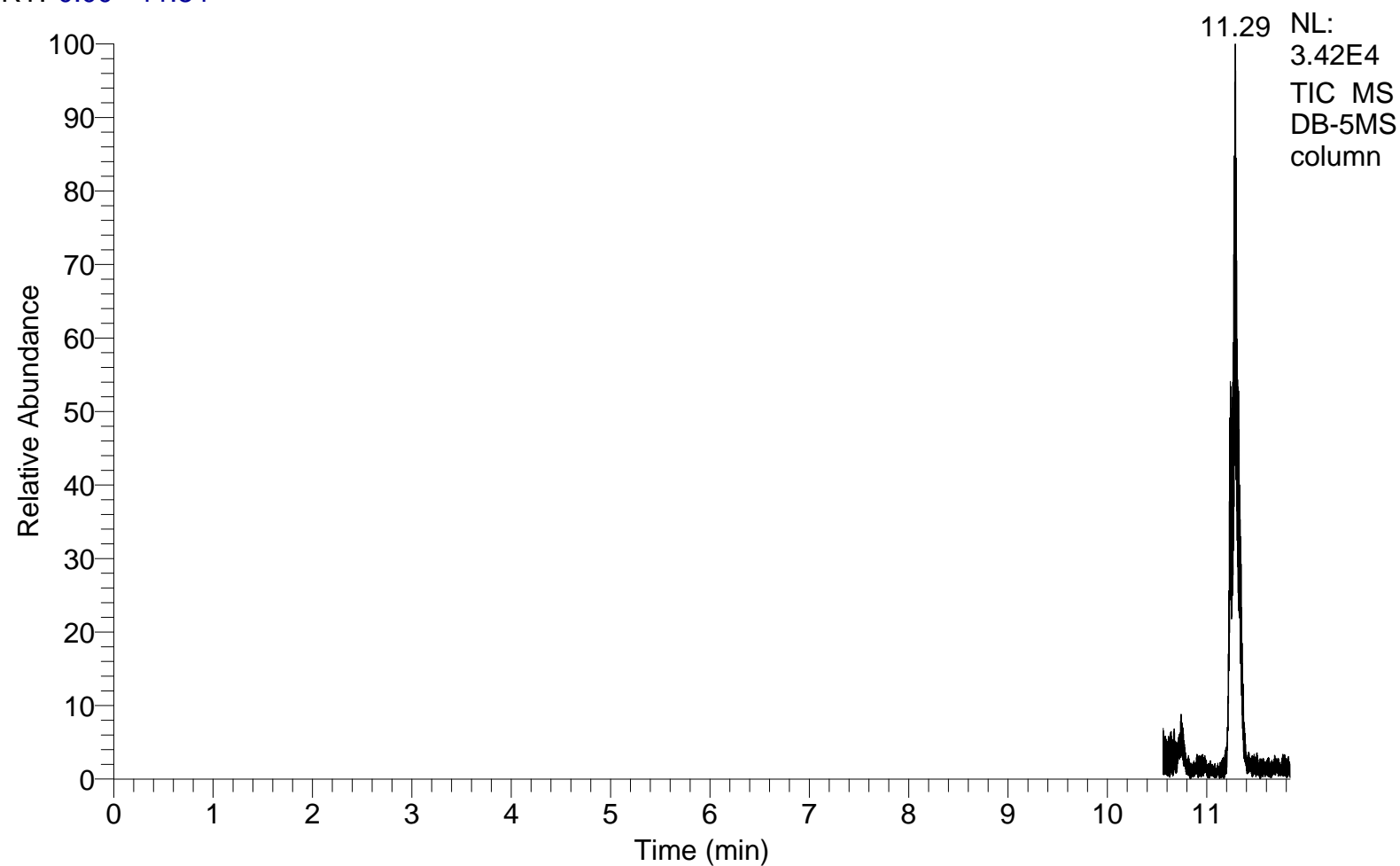

DB-5MS column #1 RT: 10.56 AV: 1 NL: 2.37E3  
T: + c EI SRM ms2 383.300@cid20.00 [170.995-171.005]

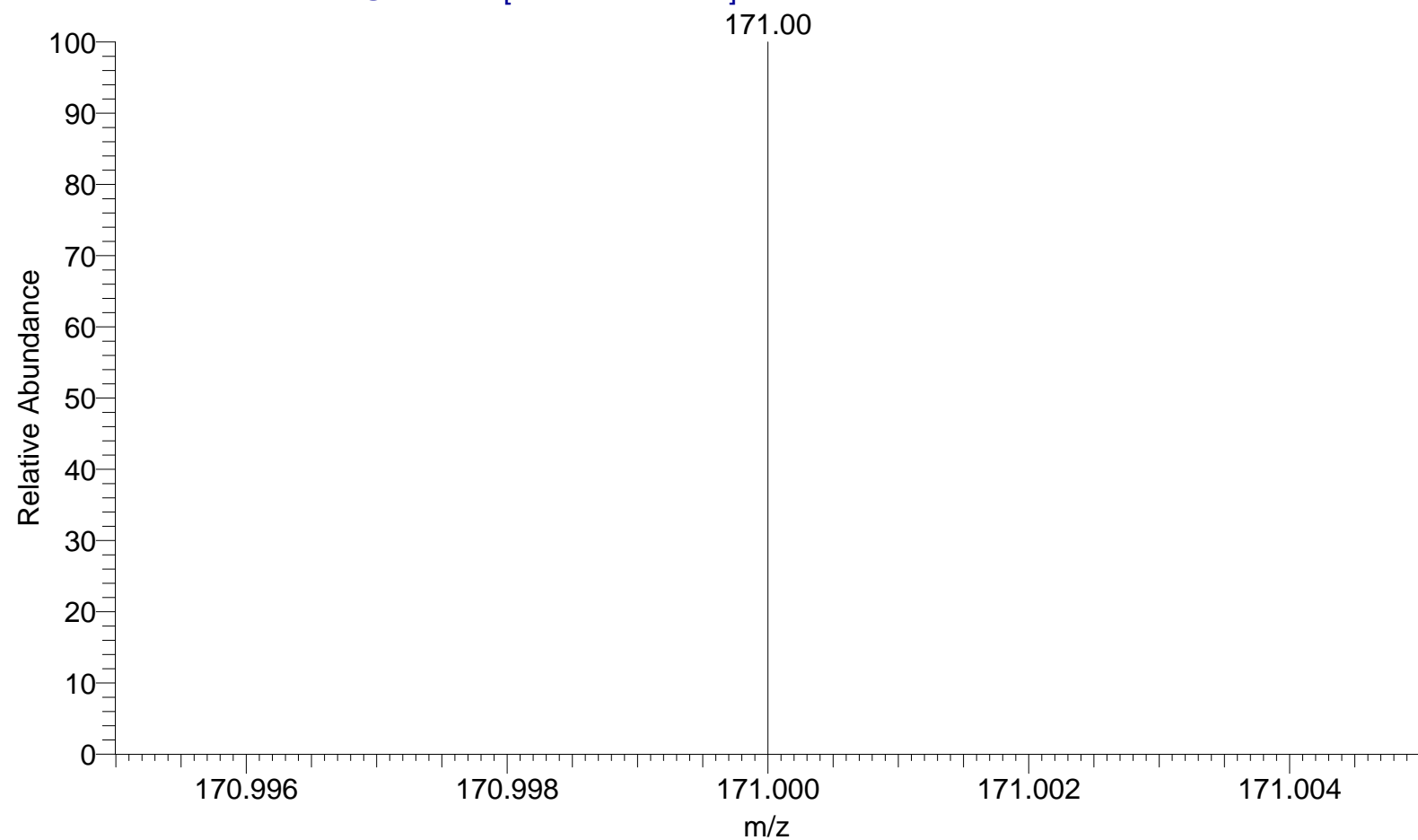

Supplement: Supplementary data 1 [file mmc1.pdf]

RT: 0.00 - 11.84

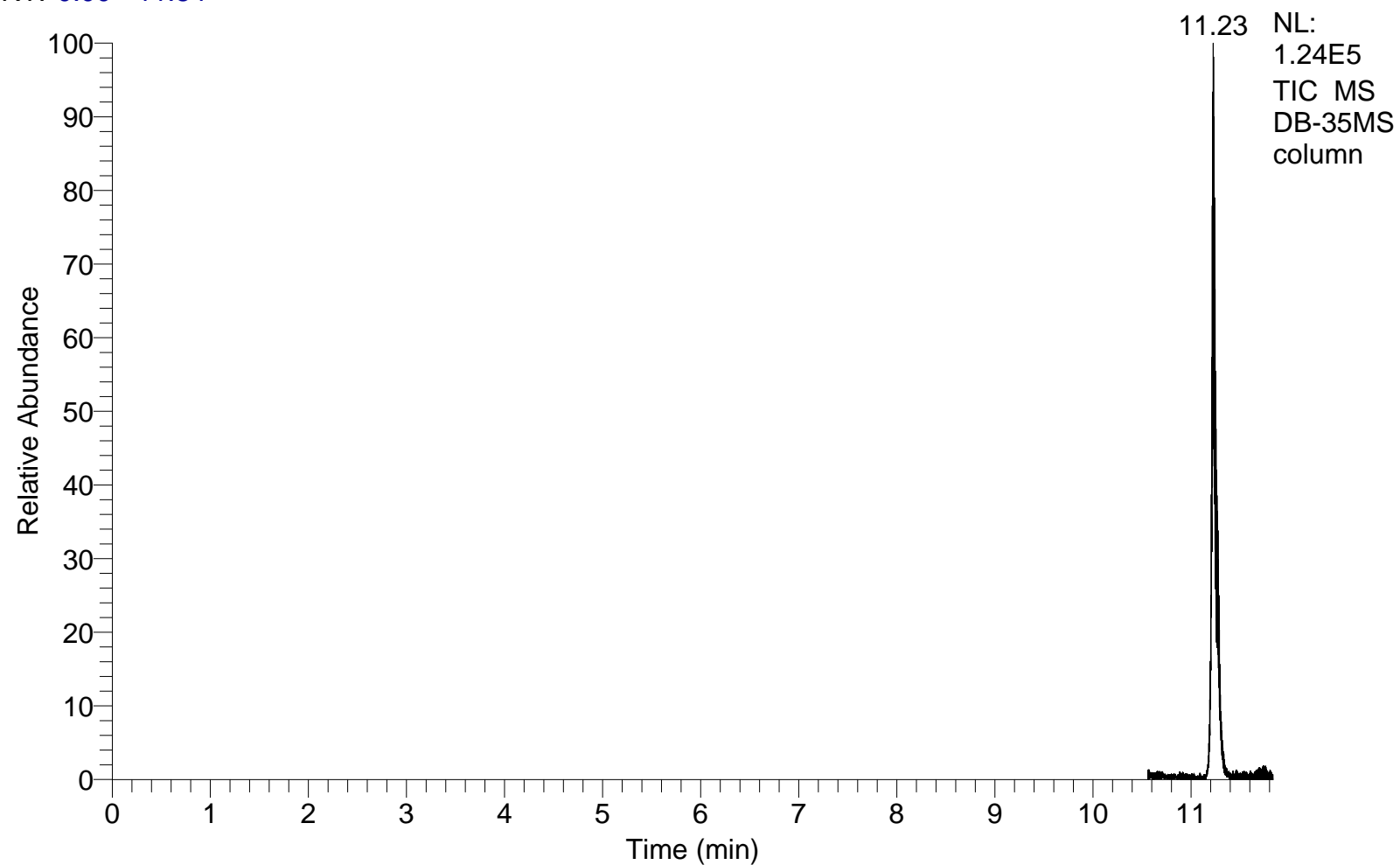

DB-35MS column #1 RT: 10.56 AV: 1 NL: 1.62E3  
T: + c EI SRM ms2 383.300@cid20.00 [170.995-171.005]

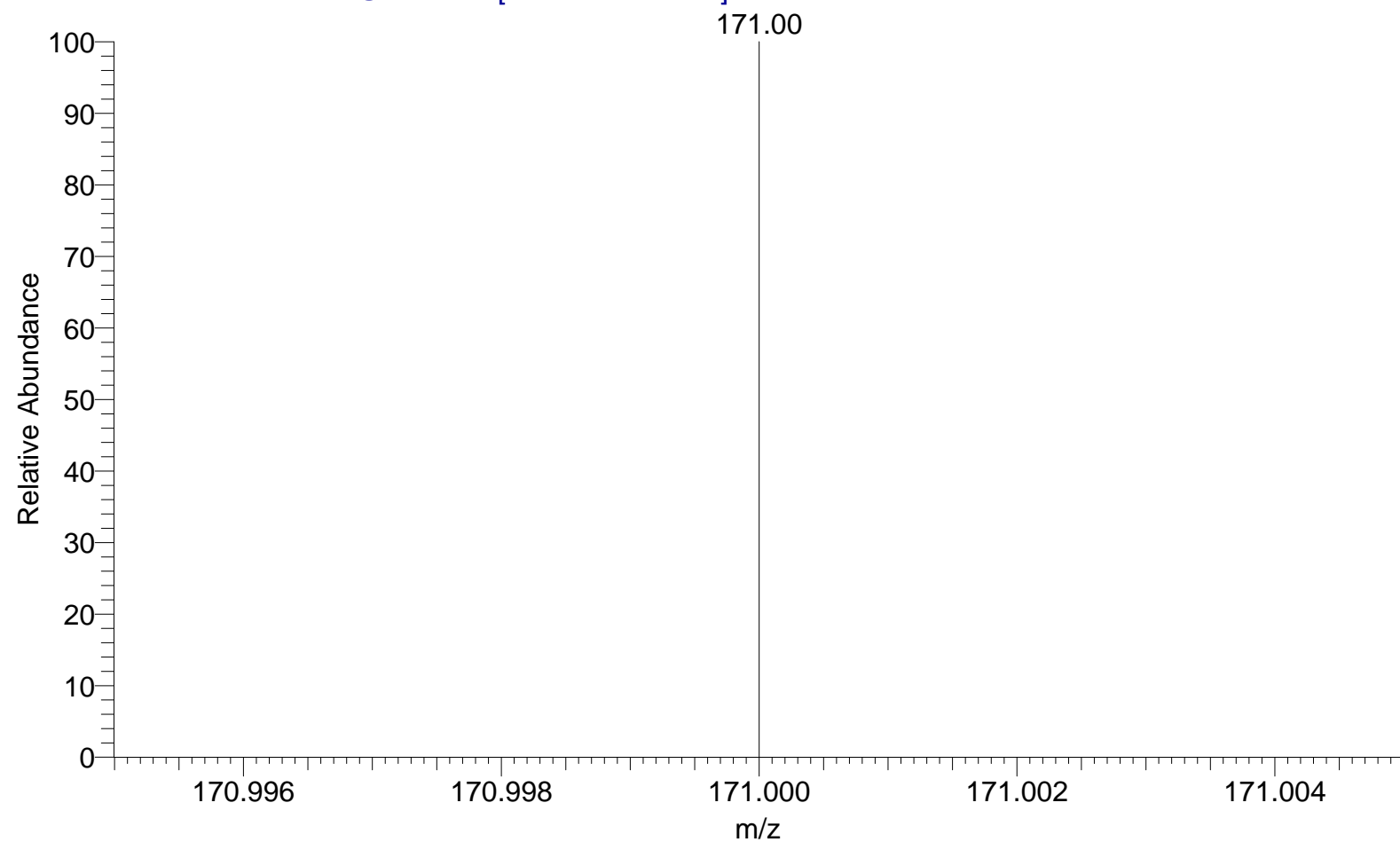

Supplement: Supplementary data 2 [file mmc2.pdf]

RT: 0.00 - 11.84

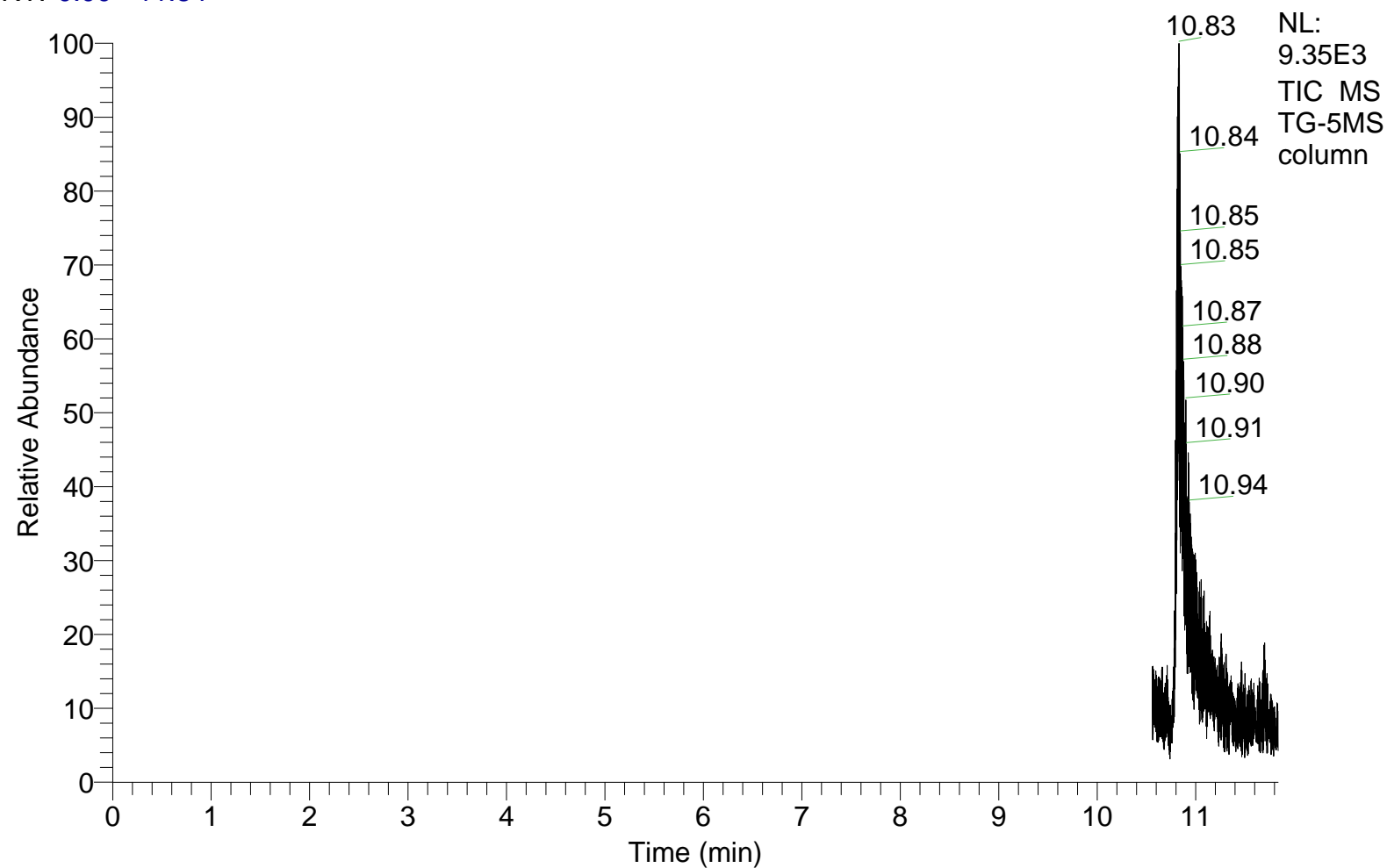

TG-5MS column #1 RT: 10.56 AV: 1 NL: 1.46E3  
T: + c EI SRM ms2 383.300@cid20.00 [170.995-171.005]

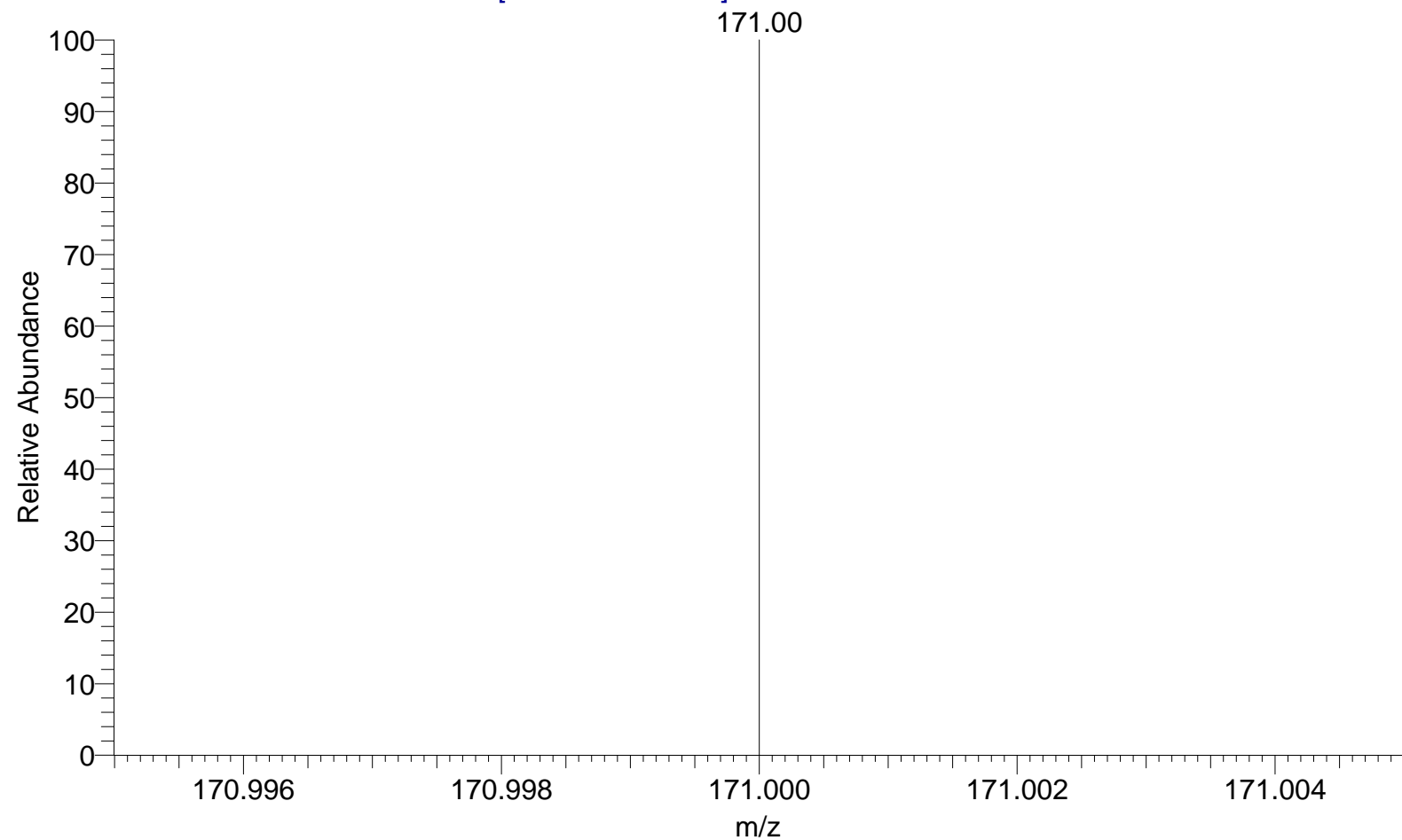

Supplement: Supplementary data 3 [file mmc3.pdf]

RT: 0.00 - 11.84

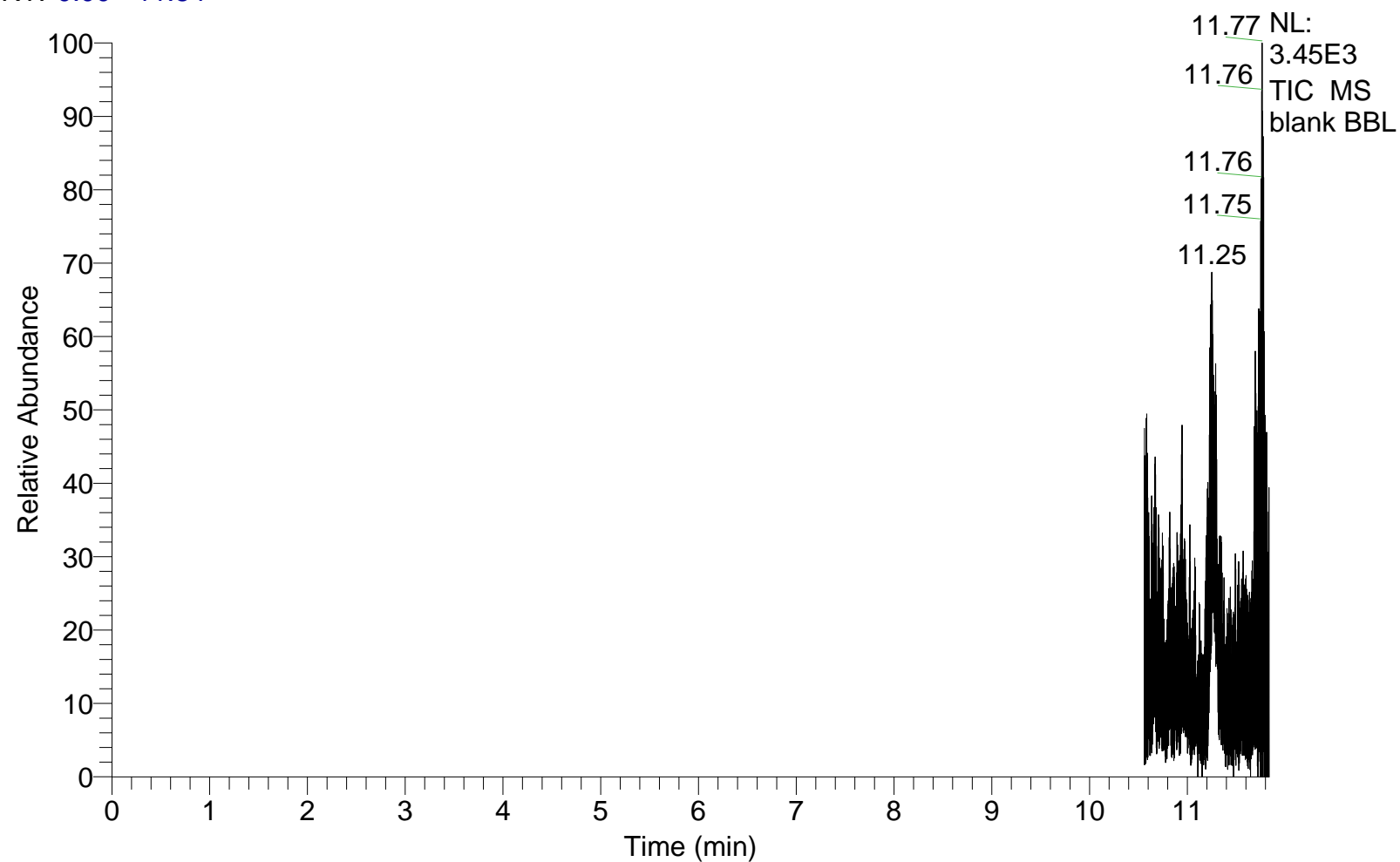

blank BBL #1 RT: 10.56 AV: 1 NL: 1.64E3  
T: + c EI SRM ms2 383.300@cid20.00 [170.995-171.005]

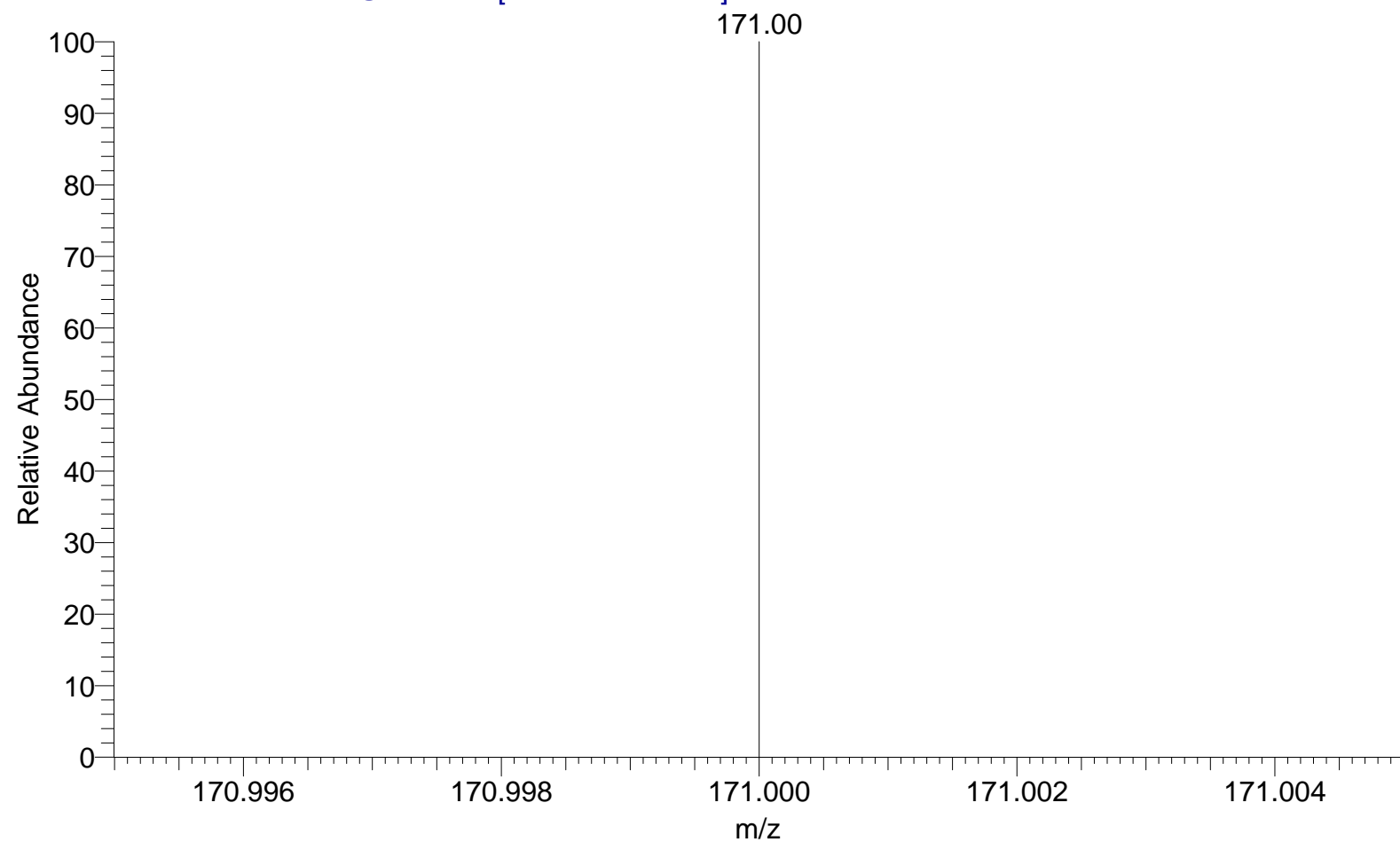

Supplement: Supplementary data 4 [file mmc4.pdf]

RT: 0.00 - 11.84

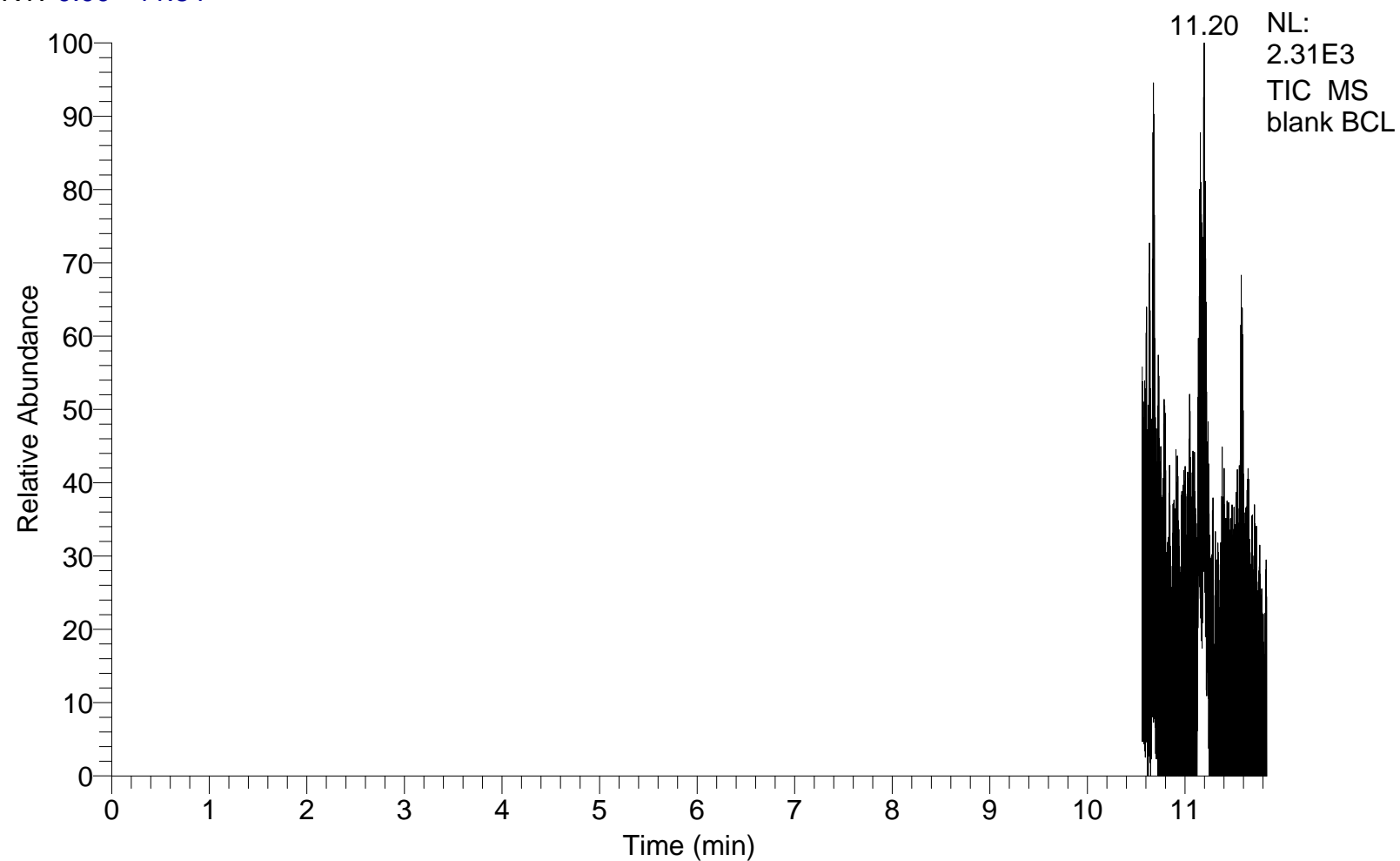

blank BCL #1 RT: 10.56 AV: 1 NL: 1.29E3  
T: + c EI SRM ms2 383.300@cid20.00 [170.995-171.005]

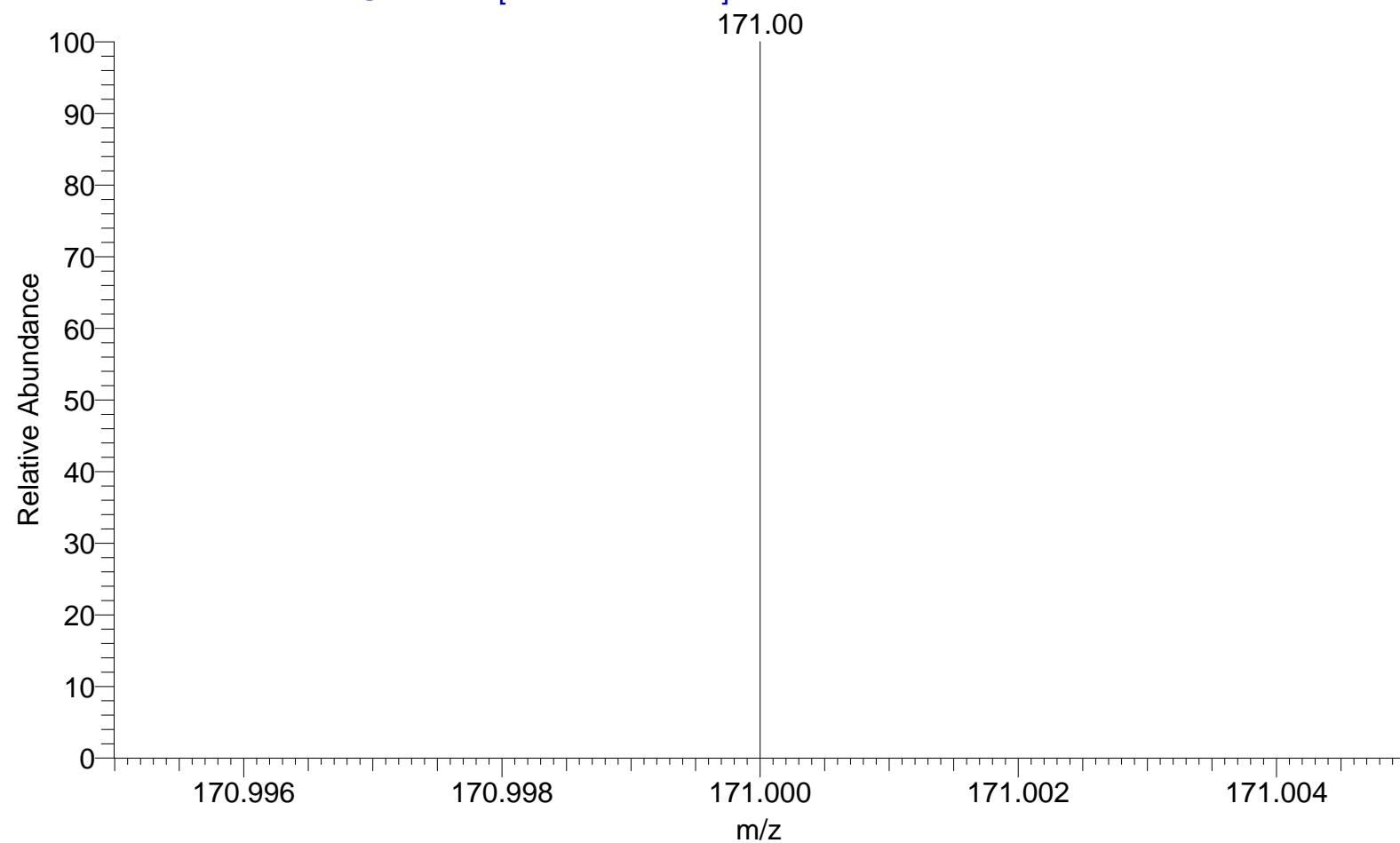

Supplement: Supplementary data 5 [file mmc5.pdf]

RT: 0.00 - 11.84

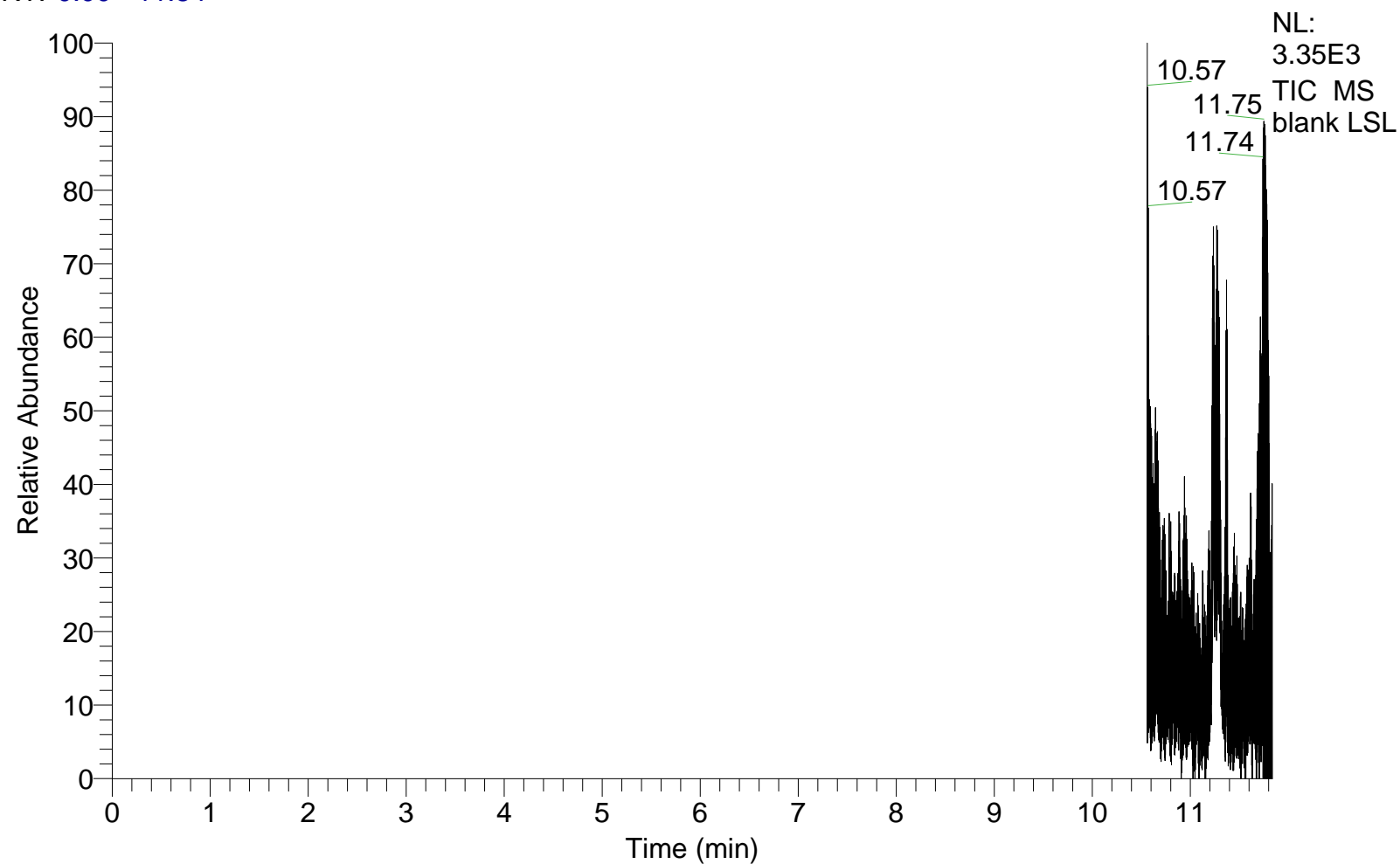

blank LSL #1 RT: 10.56 AV: 1 NL: 3.35E3  
T: + c EI SRM ms2 383.300@cid20.00 [170.995-171.005]

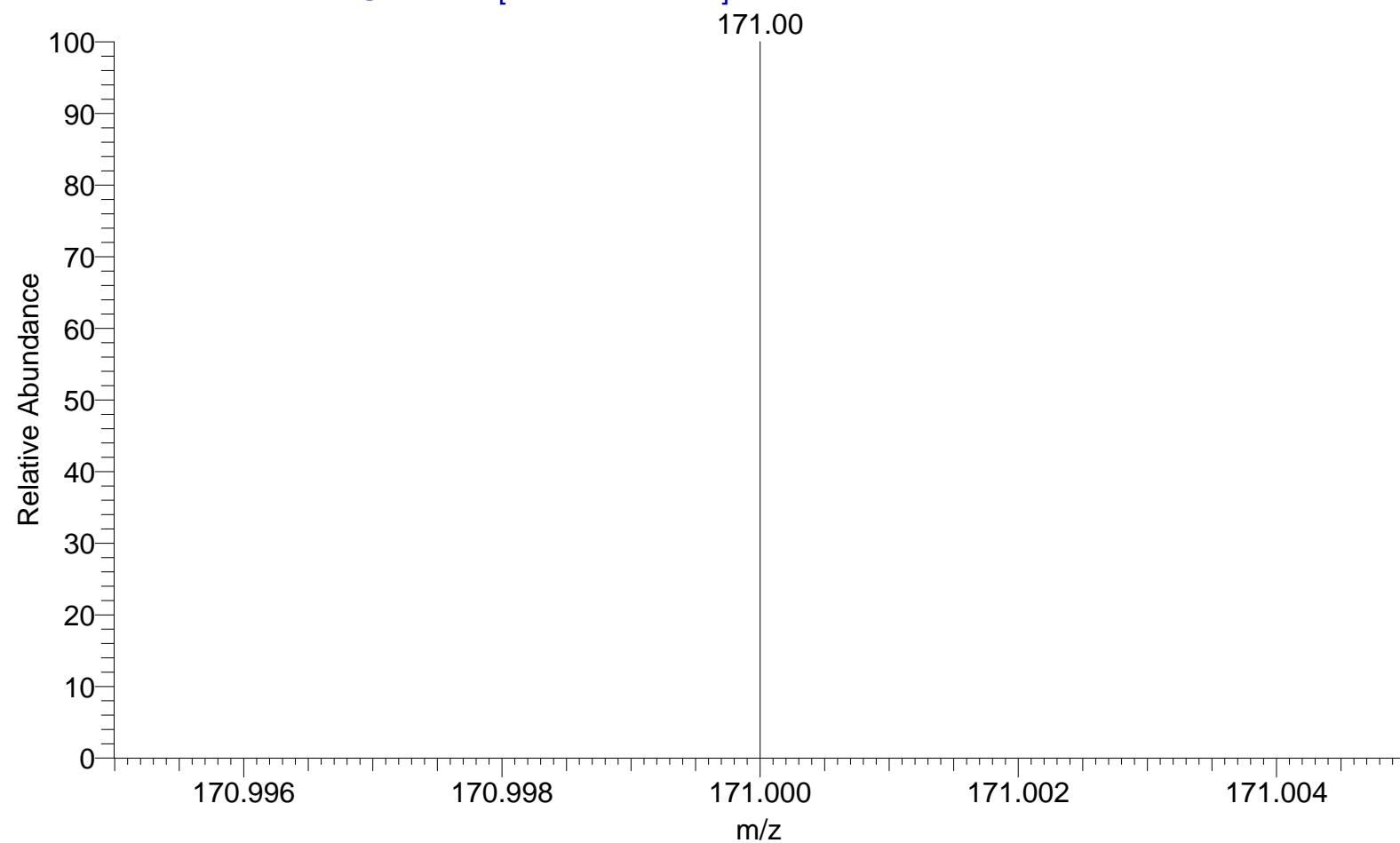

Supplement: Supplementary data 6 [file mmc6.pdf]

RT: 0.00 - 11.84

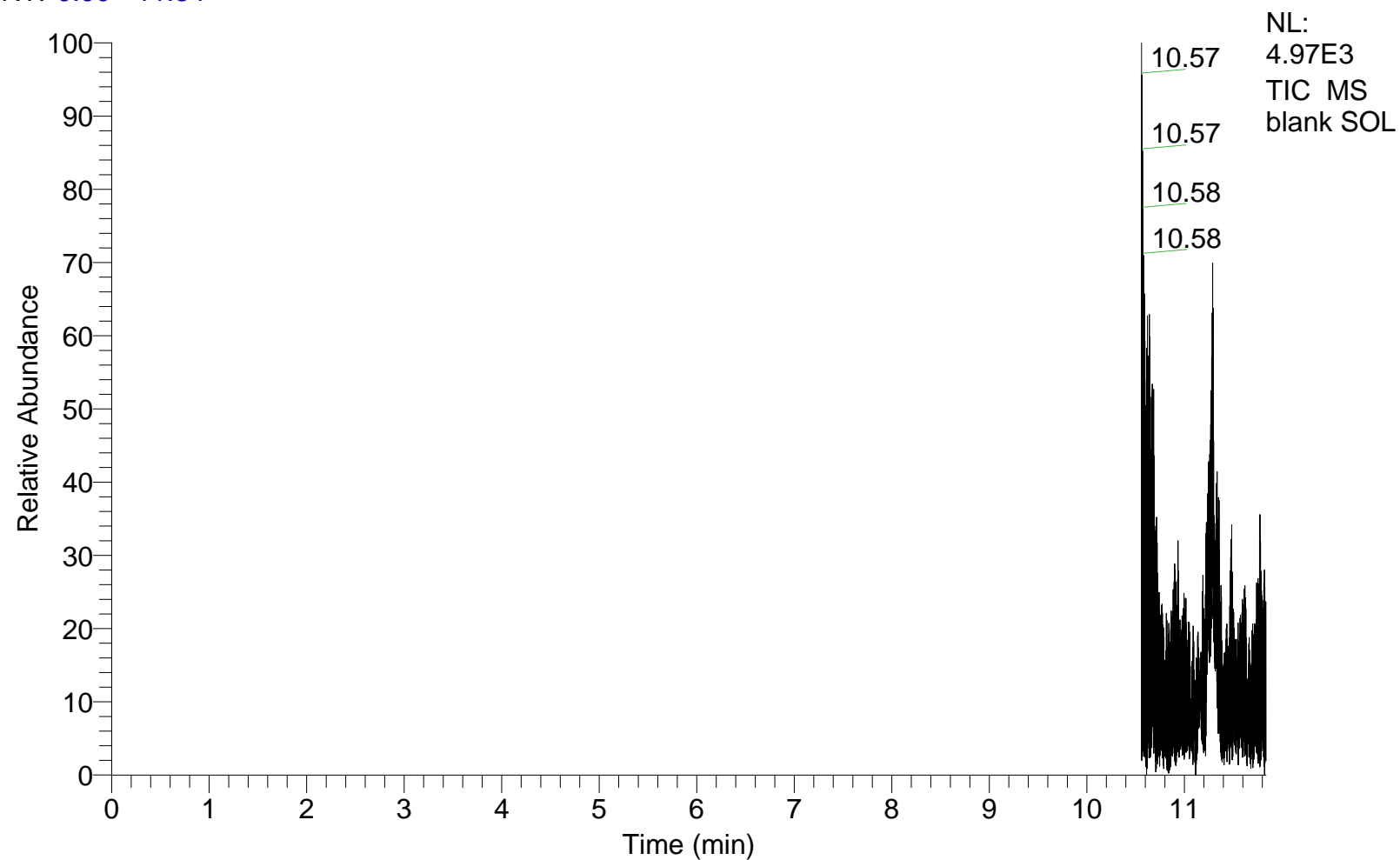

blank SOL #1 RT: 10.56 AV: 1 NL: 4.97E3  
T: + c EI SRM ms2 383.300@cid20.00 [170.995-171.005]

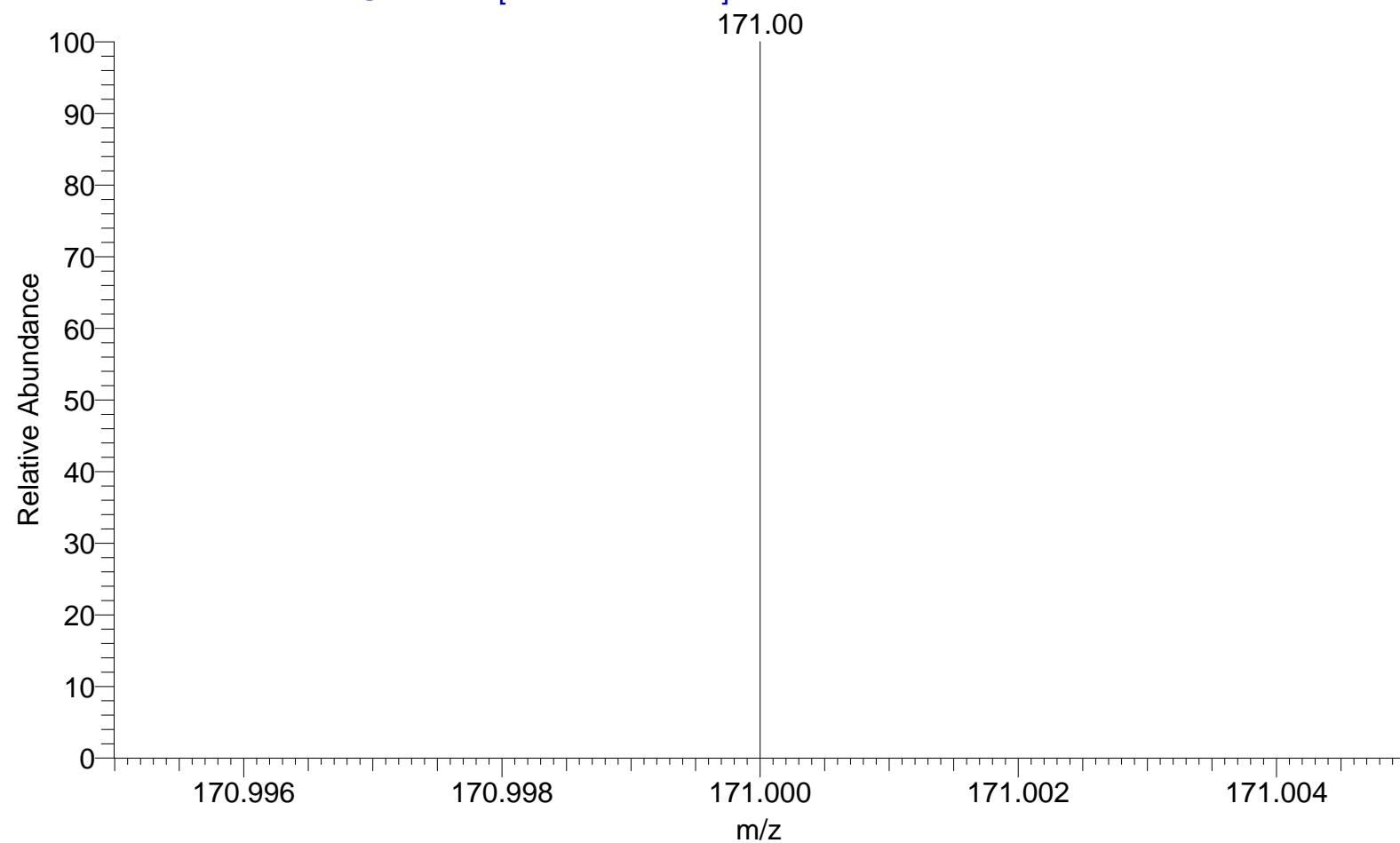

Supplement: Supplementary data 7 [file mmc7.pdf]

RT: 0.00 - 13.46

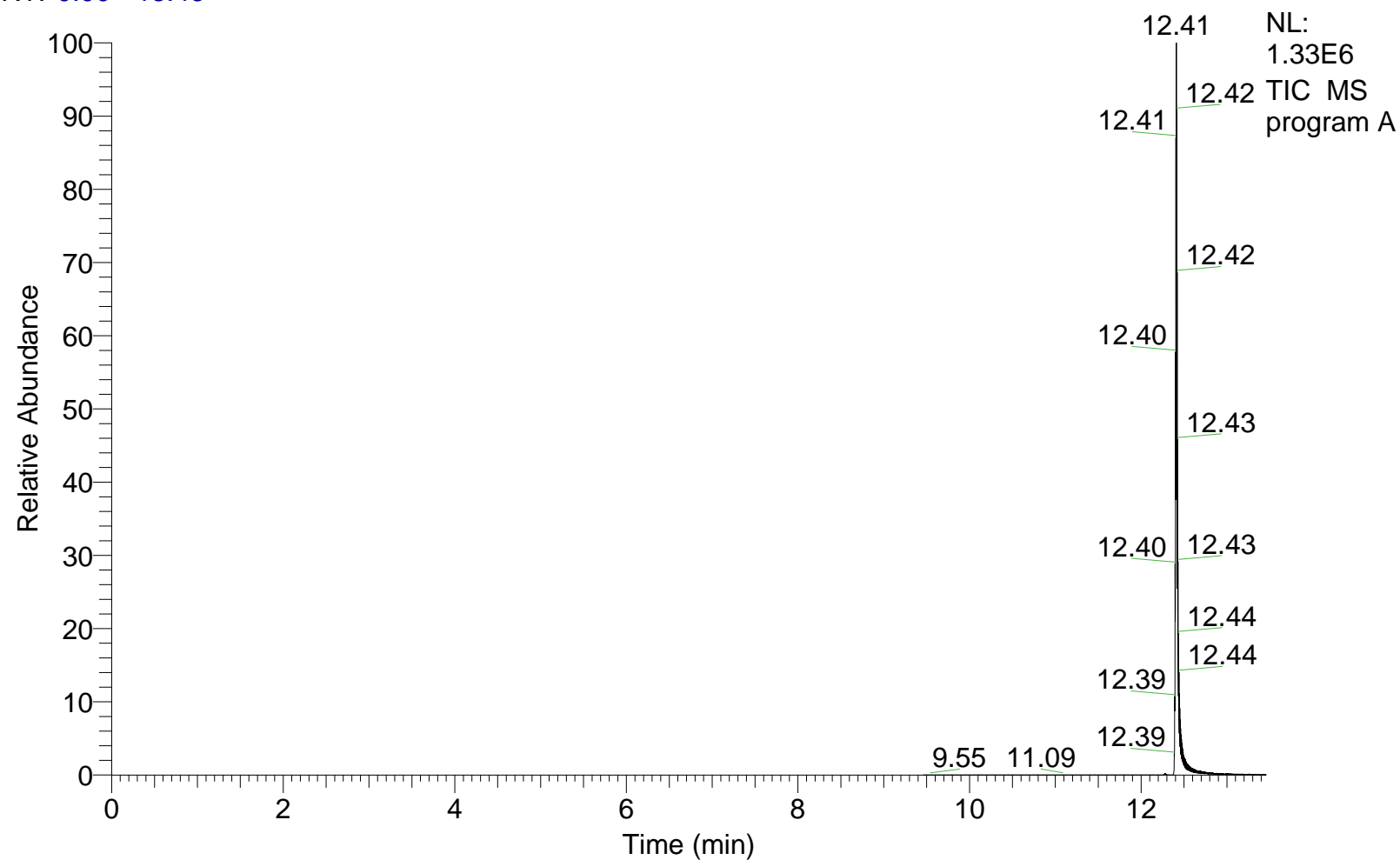

program A #1771 RT: 12.41 AV: 1 NL: 1.33E6  
T: + c EI SRM ms2 383.300@cid20.00 [170.995-171.005]

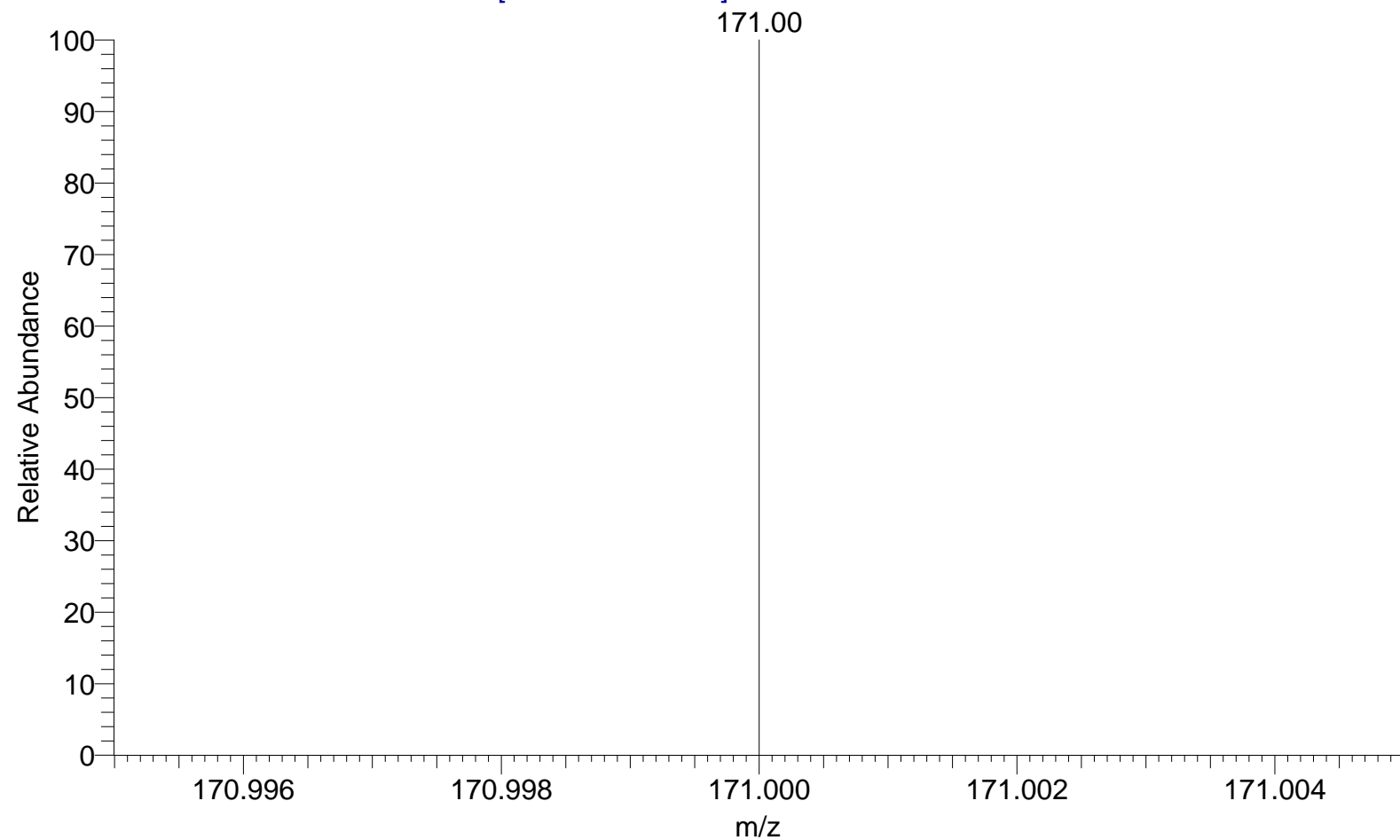

Supplement: Supplementary data 8 [file mmc8.pdf]

RT: 0.00 - 11.84

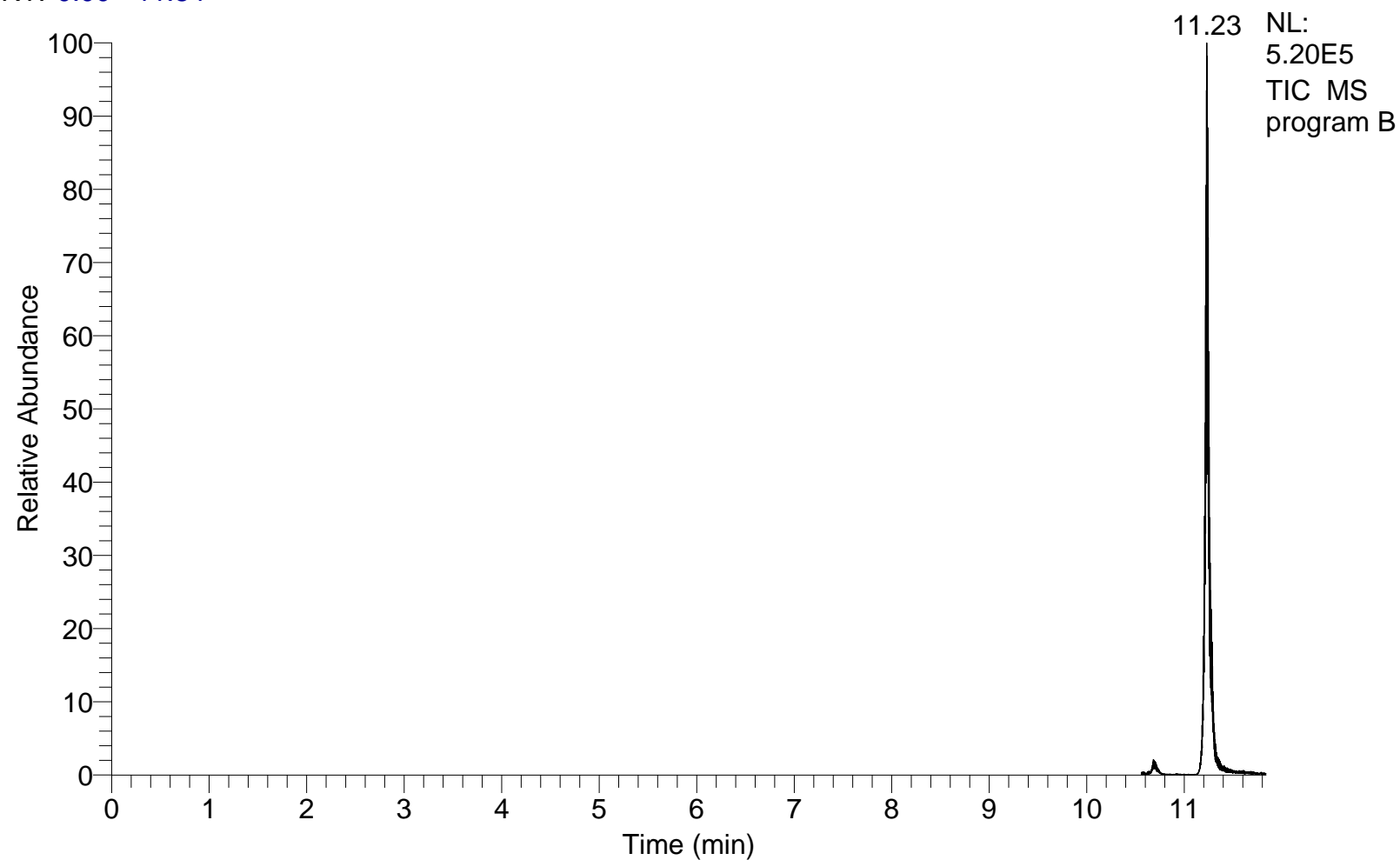

program B #1 RT: 10.56 AV: 1 NL: 2.02E3  
T: + c EI SRM ms2 383.300@cid20.00 [170.995-171.005]

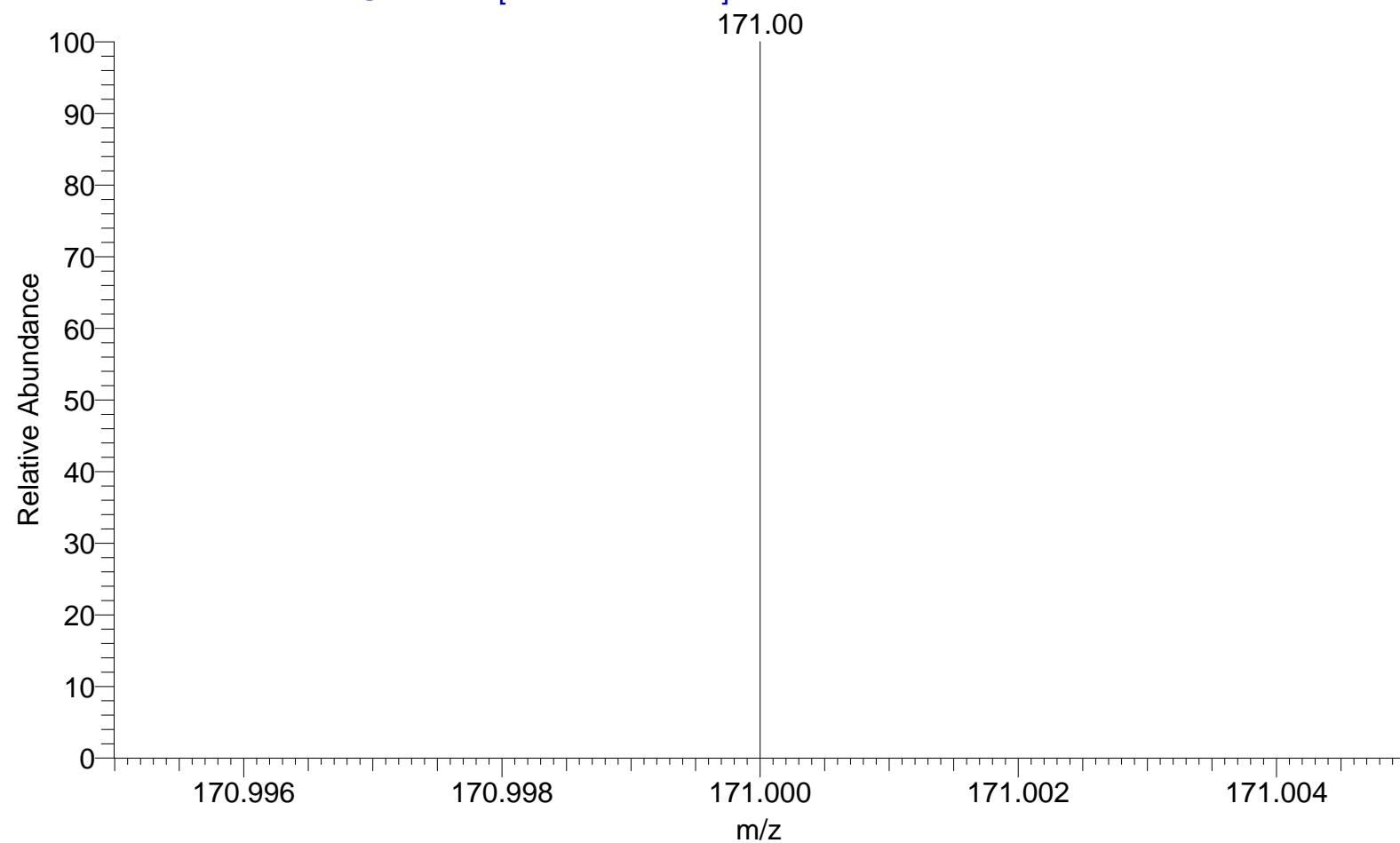

Supplement: Supplementary data 9 [file mmc9.pdf]

RT: 0.00 - 11.84

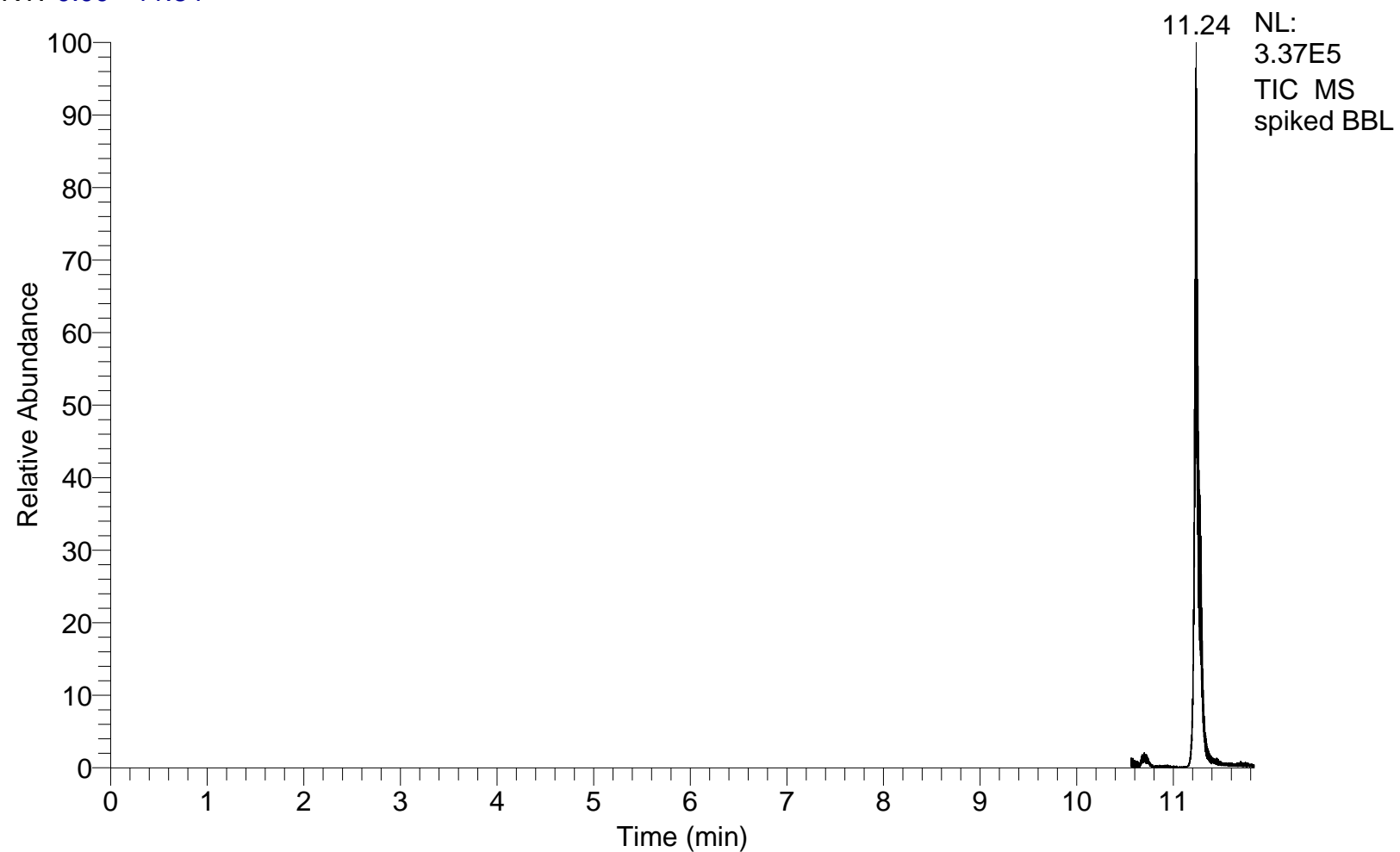

spiked BBL #1 RT: 10.56 AV: 1 NL: 4.62E3  
T: + c EI SRM ms2 383.300@cid20.00 [170.995-171.005]

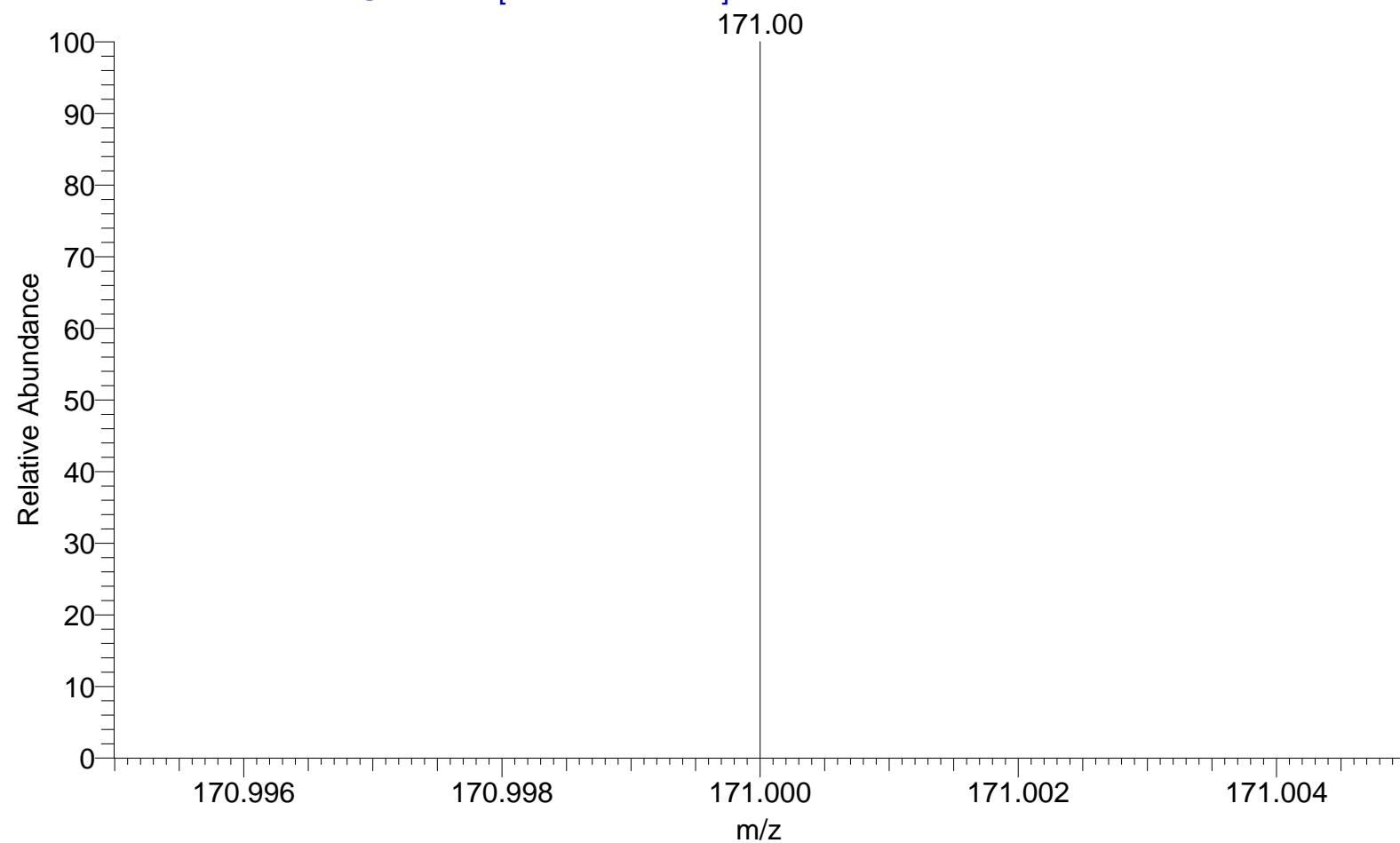

Supplement: Supplementary data 10 [file mmc10.pdf]

RT: 0.00 - 11.84

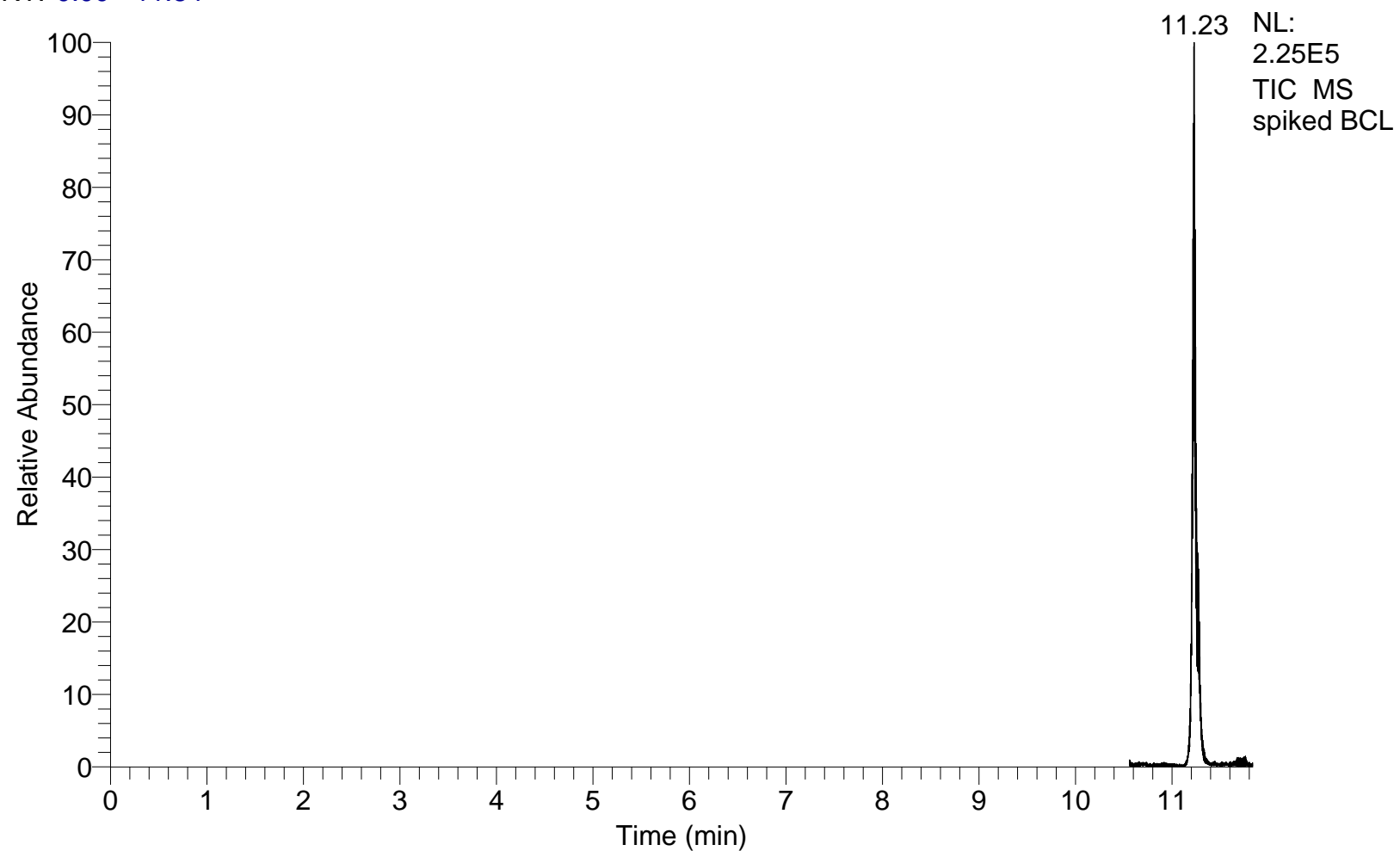

spiked BCL #1 RT: 10.56 AV: 1 NL: 2.24E3  
T: + c EI SRM ms2 383.300@cid20.00 [170.995-171.005]

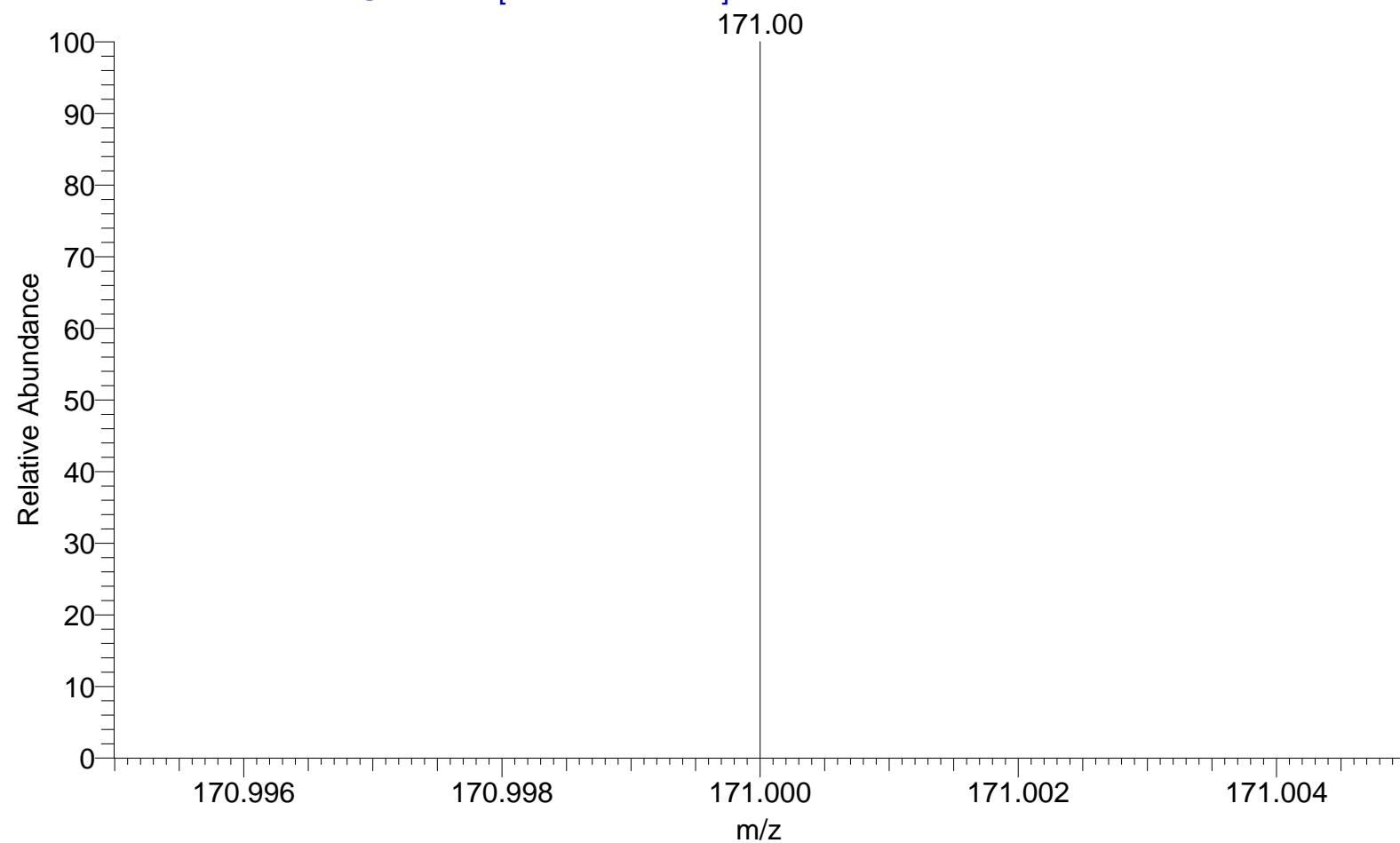

Supplement: Supplementary data 11 [file mmc11.pdf]

RT: 0.00 - 11.84

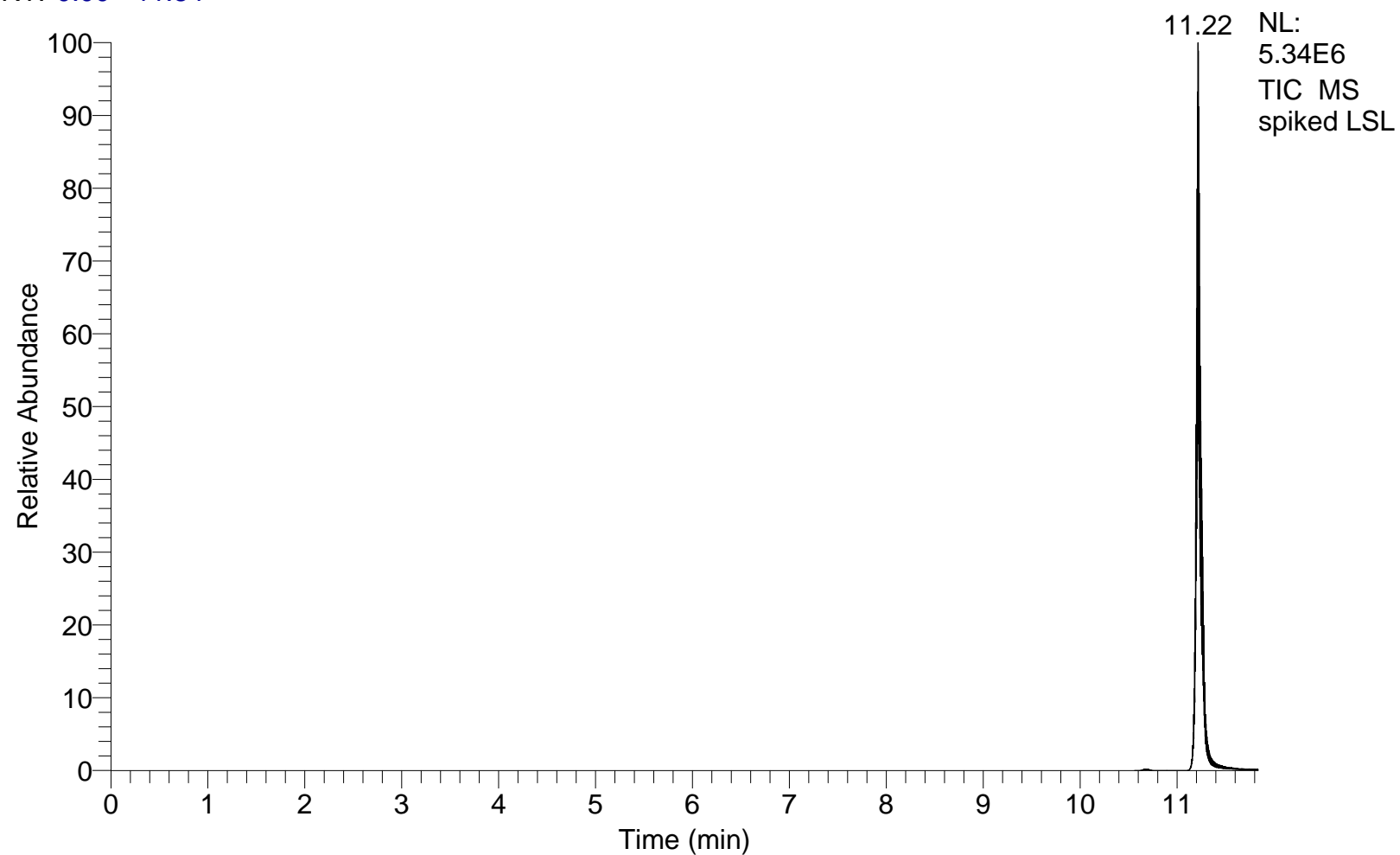

spiked LSL #1 RT: 10.56 AV: 1 NL: 7.24E1  
T: + c EI SRM ms2 383.300@cid20.00 [170.995-171.005]

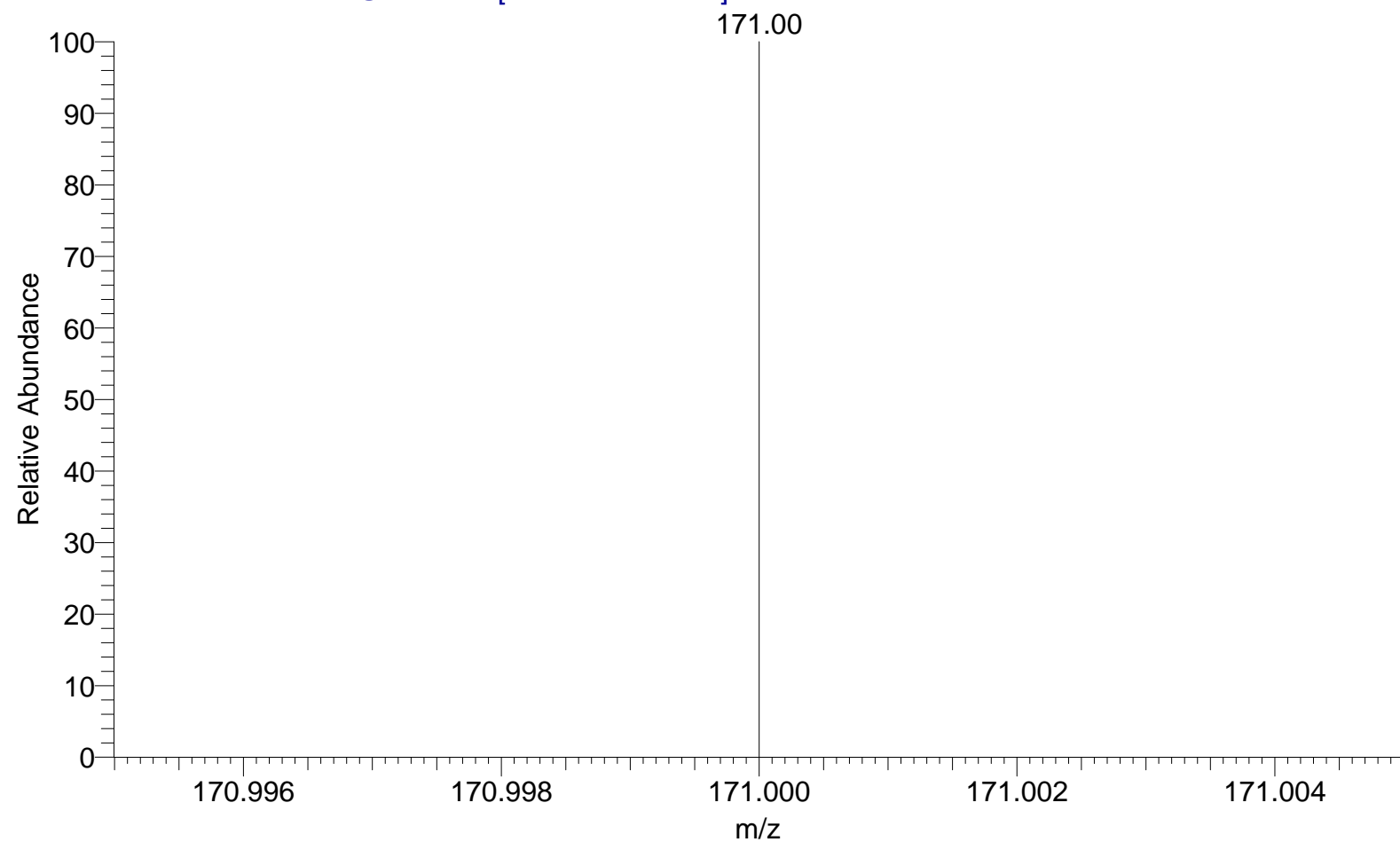

Supplement: Supplementary data 12 [file mmc12.pdf]

RT: 0.00 - 11.84

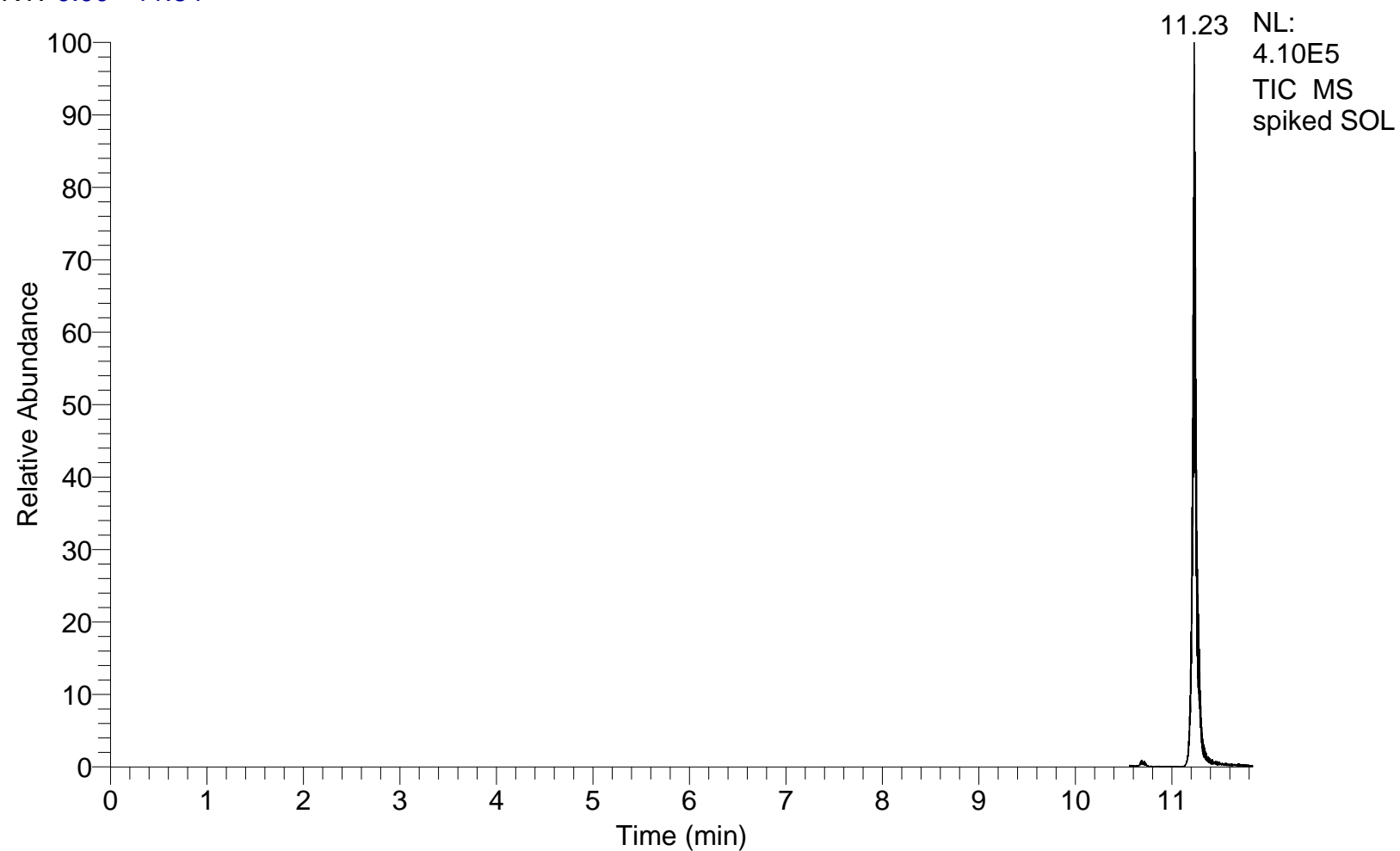

spiked SOL #1 RT: 10.56 AV: 1 NL: 1.03E3  
T: + c EI SRM ms2 383.300@cid20.00 [170.995-171.005]

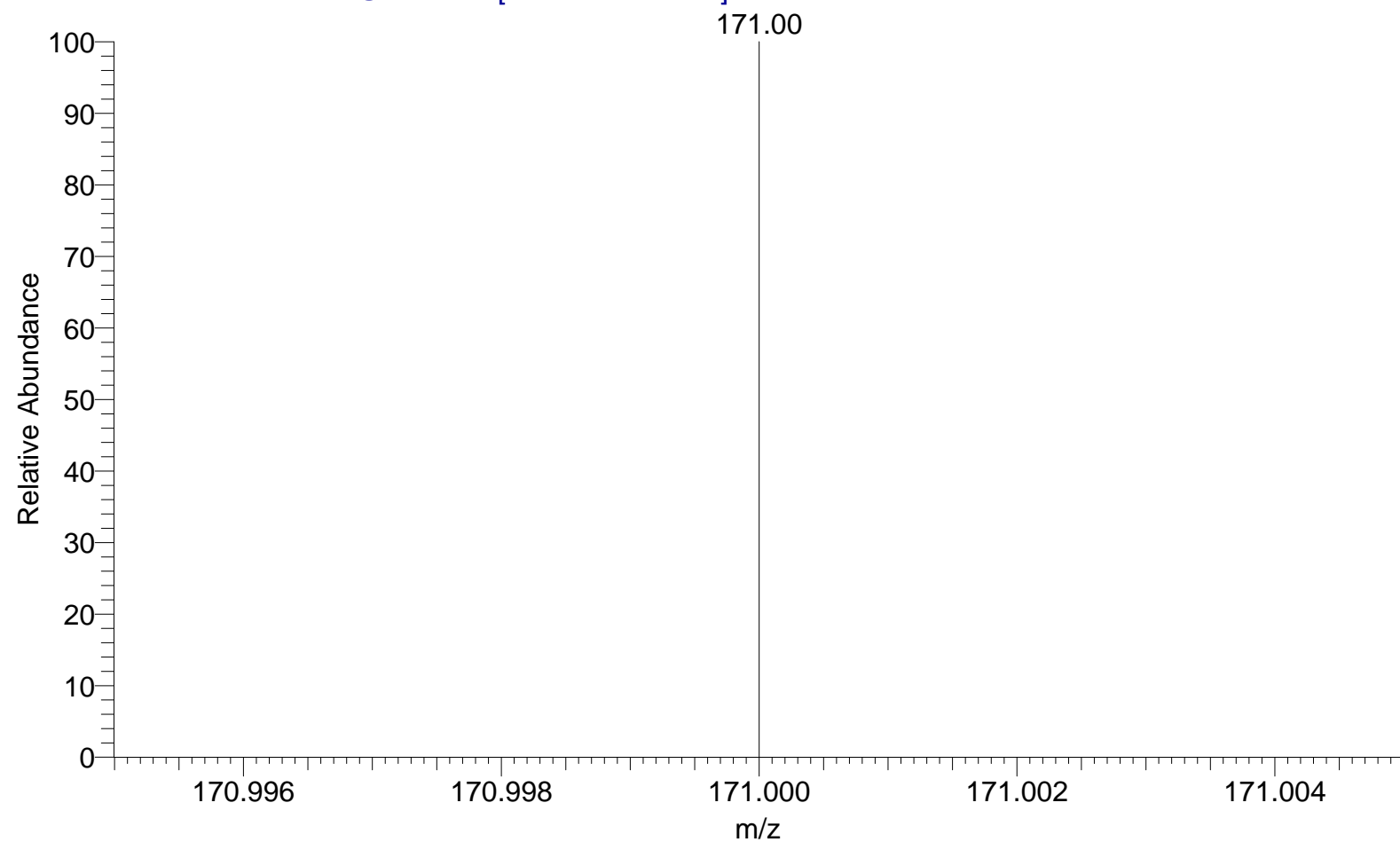

Supplement: Supplementary data 13 [file mmc13.pdf]

RT: 0.00 - 11.84

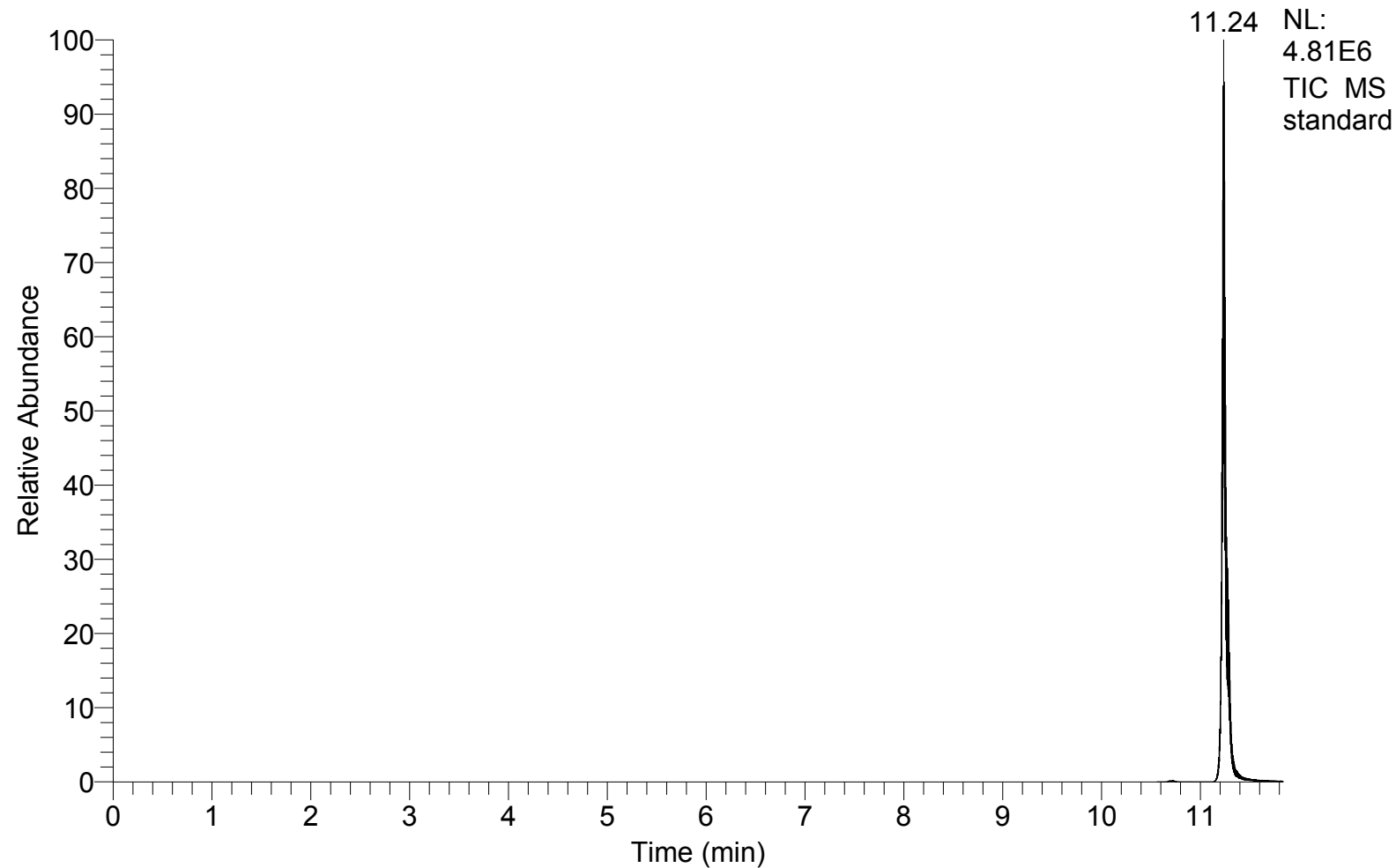

standard #1 RT: 10.56 AV: 1 NL: 7.01E2  
T: + c EI SRM ms2 383.300@cid20.00 [170.995-171.005]

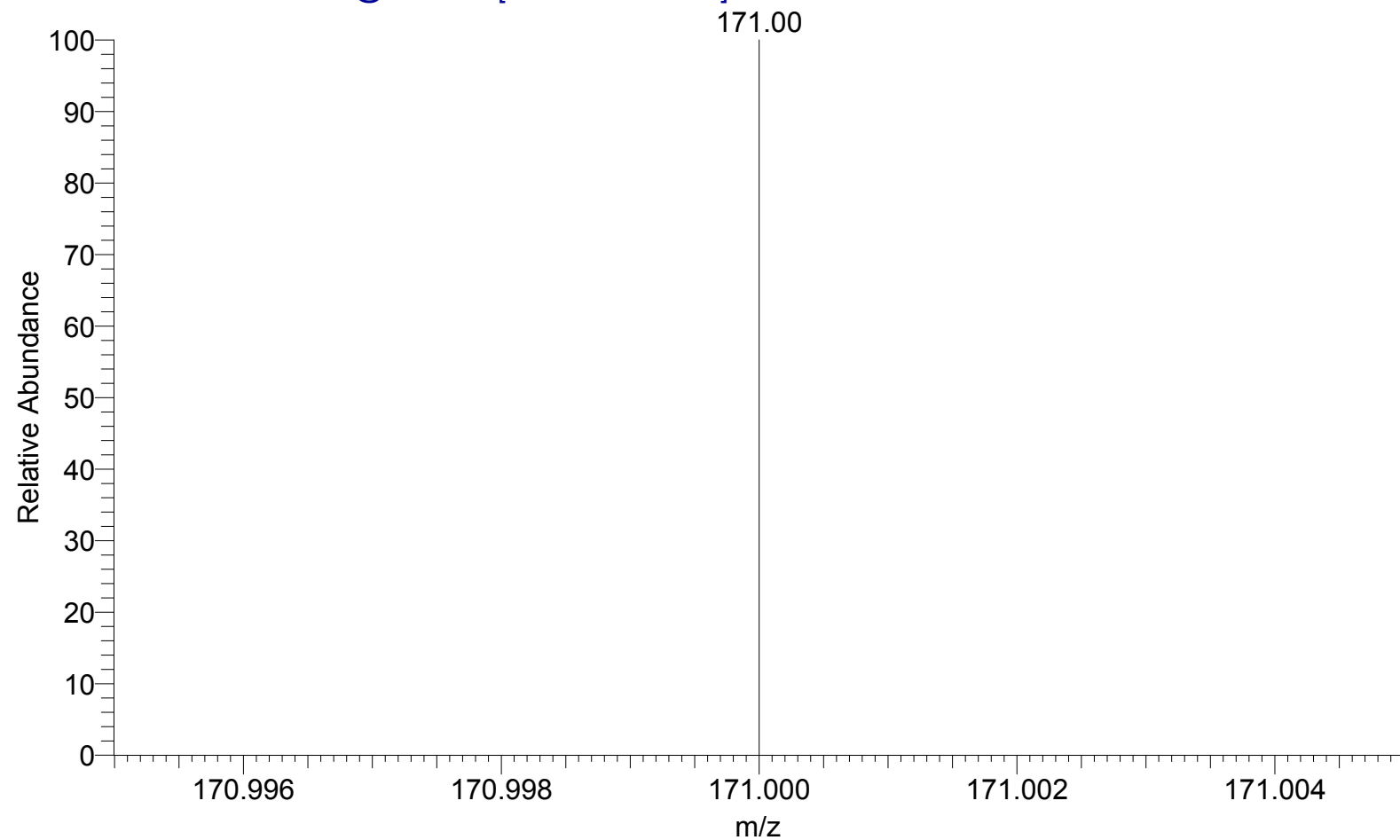

Supplement: Supplementary data 14 [file mmc14.pdf]
