# Supplementary material for: Residue, dissipation and dietary intake risk assessment of tolfenpyrad in four leafy green vegetables under greenhouse conditions
Source: Food Chem X. 2022 Feb 4;13:100241. doi: 10.1016/j.fochx.2022.100241 (PMC9040032; doi:10.1016/j.fochx.2022.100241)
Supplement: Supplementary data 15 [file mmc15.docx]

**Supplementary information**

**Fig. S1.** GC-MS/MS chromatograms of tolfenpyrad in standard solution (0.05 μg/mL) on different types of chromatographic column. DB-35MS represents Agilent J&W DB-35ms GC column (30 m × 0.25 mm, 0.25 µm), DB-5MS represents Agilent J&W DB-5ms GC column (30 m × 0.25 mm, 0.25 µm), and TG-5MS represents Thermo Scientific^TM^ TraceGOLD^TM^ TG-5MS GC column (30 m × 0.25 mm, 0.25 µm).

**Fig. S2.** GC-MS/MS chromatograms of tolfenpyrad in standard solution (0.05 μg/mL) on Agilent J&W DB-35ms column (30 m × 0.25 mm, 0.25 µm) with different temperature programs, A represents the temperature program (the initial temperature was set at 100 °C, held for 0.5 min, increased to 220 °C at 20 °C/min, held for 2 min, increased to 300 °C at 40 °C/min and held for 5 min) and B represents the temperature program (the initial temperature was set at 100 °C, held for 0.5 min, increased to 220 °C at 20 °C/min, held for 1 min, increased to 300 °C at 40 °C/min and held for 3 min).

**Fig. S3.** Extraction efficiency of tolfenpyrad from different leafy green vegetables with different extraction methods and times (spiked at 0.1 mg/kg). Error bars represent standard deviation (SD) of triplicates. In each leafy vegetable, columns marked by different case letters are significantly different from each other and *P* value is evaluated by Duncan’s multiple range test (*P* < 0.01). BBL: *Brassica bara* L.; SOL: *Spinacia oleracea* L.; LSL: *Lactuca sativa* L.; and BCL: *Brassica chinensis* L.

**Fig. S4.** Extraction efficiency of tolfenpyrad from different leafy green vegetables with different extraction solvents (spiked at 0.1 mg/kg). Error bars represent standard deviation (SD) of triplicates. In each leafy vegetable, columns marked by different case letters are significantly different from each other and *P* value is evaluated by Duncan’s multiple range test (*P* < 0.01). BBL: *Brassica bara* L.; SOL: *Spinacia oleracea* L.; LSL: *Lactuca sativa* L.; and BCL: *Brassica chinensis* L.

**Fig. S5.** Extraction efficiency of tolfenpyrad from different leafy green vegetables with different volumes of extractant (spiked at 0.1 mg/kg). Error bars represent standard deviation (SD) of triplicates. In each leafy vegetable, columns marked by different case letters are significantly different from each other and *P* value is evaluated by Duncan’s multiple range test (*P* < 0.01). BBL: *Brassica bara* L.; SOL: *Spinacia oleracea* L.; LSL: *Lactuca sativa* L.; and BCL: *Brassica chinensis* L.

**Fig. S6.** Extraction efficiency of tolfenpyrad from different leafy green vegetables with different sorbents (spiked at 0.1 mg/kg). Sorbent 1: 50 mg C_18_, sorbent 2: 100 mg C_18_, sorbent 3: 50 mg PSA, sorbent 4: 100 mg PSA, sorbent 5: 50 mg GCB, sorbent 6: 100 mg GCB, sorbent 7: 50 mg C_18_+50 mg PSA. Error bars represent standard deviation (SD) of triplicates. In each leafy vegetable, columns marked by different case letters are significantly different from each other and *P* value is evaluated by Duncan’s multiple range test (*P* < 0.01). BBL: *Brassica bara* L.; SOL: *Spinacia oleracea* L.; LSL: *Lactuca sativa* L.; and BCL: *Brassica chinensis* L.

**Fig. S7.** Residue levels and dissipation rates of tolfenpyrad in *Brassica bara* L. samples collected at different intervals with several spraying doses and times. Error bars represent standard deviation (SD) of triplicates. Different case letters indicate significant differences between the ten sampling intervals (*P* < 0.01, Duncan’s multiple range test). A, B, C and D represent spraying dose of 67.5 g a.i./ha once, spraying dose of 67.5 g a.i./ha twice, spraying dose of 112.5 g a.i./ha once and spraying dose of 112.5 g a.i./ha twice.

**Fig. S8.** Residue levels and dissipation rates of tolfenpyrad in *Spinacia oleracea* L.samples collected at different intervals with several spraying doses and times. Error bars represent standard deviation (SD) of triplicates. Different case letters indicate significant differences between the ten sampling intervals (*P* < 0.01, Duncan’s multiple range test). A, B, C and D represent spraying dose of 67.5 g a.i./ha once, spraying dose of 67.5 g a.i./ha twice, spraying dose of 112.5 g a.i./ha once and spraying dose of 112.5 g a.i./ha twice.

**Fig. S9.** Residue levels and dissipation rates of tolfenpyrad in *Lactuca sativa* L. samples collected at different intervals with several spraying doses and times. Error bars represent standard deviation (SD) of triplicates. Different case letters indicate significant differences between the ten sampling intervals (*P* < 0.01, Duncan’s multiple range test). A, B, C and D represent spraying dose of 67.5 g a.i./ha once, spraying dose of 67.5 g a.i./ha twice, spraying dose of 112.5 g a.i./ha once and spraying dose of 112.5 g a.i./ha twice.

**Fig. S10.** Residue levels and dissipation rates of tolfenpyrad in *Brassica chinensis* L. samples collected at different intervals with several spraying doses and times. Error bars represent standard deviation (SD) of triplicates. Different case letters indicate significant differences between the ten sampling intervals (*P* < 0.01, Duncan’s multiple range test). A, B, C and D represent spraying dose of 67.5 g a.i./ha once, spraying dose of 67.5 g a.i./ha twice, spraying dose of 112.5 g a.i./ha once and spraying dose of 112.5 g a.i./ha twice.

**Table S1** MS/MS parameters of tolfenpyrad.

| Ion source | Collision gas | Monitoring mode | Parent ion (*m*/*z*) | Product ion (*m/z*) | CE (eV) |
| --- | --- | --- | --- | --- | --- |
| Electron impact  (EI) | Argon  (99.999%) | Selected reaction monitoring  (SRM) | 383.3 | 171.1 (Quantitation) | 20 |
|  |  |  |  | 181.1 (Confirmation) | 10 |
|  |  |  |  | 197.1 (Confirmation) | 20 |

**Table S2** Linear equation, determination coefficient (*R*^2^), LOD, LOQ and matrix effect (ME) of tolfenpyrad in solvent and different matrices.

| Matrix | Linear equation | *R*^2^ | LOD (mg/kg) | LOQ (mg/kg) | ME |
| --- | --- | --- | --- | --- | --- |
| Acetone | *y* = 4265861 *x* + 102536 | 0.9999 | / | / | / |
| BBL | *y* = 3731096 *x* + 102718 | 0.9996 | 0.003 | 0.01 | 0.87 |
| SOL | *y* = 3085044 *x* + 140846 | 0.9992 | 0.003 | 0.01 | 0.72 |
| LSL | *y* = 3321617 *x* + 153328 | 0.9994 | 0.003 | 0.01 | 0.78 |
| BCL | *y* = 3890887 *x* + 111934 | 0.9995 | 0.003 | 0.01 | 0.91 |

BBL: *Brassica bara* L.; SOL: *Spinacia oleracea* L.; LSL: *Lactuca sativa* L.; and BCL: *Brassica chinensis* L.
